# Neutron Depth Profiling: The Core Link Connecting the Microscopic Mechanisms of Lithium‐Ion Batteries With Intelligent Design

**DOI:** 10.1002/advs.78467

**Published:** 2026-11-01

**Authors:** Yulong Zhang, Chunjing Yue, Shijiao Liu, Muyang Chen, Xiaokang Shi, Rui Tan, Hongqing Hao, Jie Yang, Xinhua Liu

**Affiliations:** ^1^ College of Mechanical and Electrical Engineering Hebei Agricultural University Baoding China; ^2^ School of Computer Science University of Birmingham Birmingham UK; ^3^ School of Chemical Engineering Swansea University Swansea UK; ^4^ School of Transportation Science and Engineering Beihang University Beijing China; ^5^ Warwick Electrochemical Engineering Warwick Manufacture Group University of Warwick Coventry UK

**Keywords:** intelligent battery design, lithium dendrites, lithium‐ion battery, neutron depth profiling, solid‐state electrolyte

## Abstract

Neutron Depth Profiling (NDP) is a non‐destructive, in situ quantitative depth‐analysis technique underpinned by neutron‐induced nuclear reactions. Offering high ^6^Li‐isotope sensitivity and nanometer‐to‐micrometer depth resolution, NDP delivers isotope‐resolved lithium spatial information hardly accessible via conventional probes including secondary‐ion mass spectrometry (SIMS), X‐ray photoelectron spectroscopy (XPS), and operando neutron imaging, yet it suffers from depth‐dependent resolution degradation, limited lateral performance, and requires large‐scale neutron‐beam facilities. This paper systematically reviews NDP fundamentals, global neutron‐source progress, and cutting‐edge battery applications, covering lithium‐ion migration in electrodes, lithium dendrite nucleation and growth, and the “reversible short‐circuit” effect at solid‐electrolyte interfaces. The unique merit of NDP for quantitative ^6^Li depth‐profiling is highlighted, while noting that NDP cannot independently resolve full‐element maps or nanoscale interfaces and demands complementary characterizations. A roadmap integrating NDP with multimodal measurements and physics‐informed machine learning for intelligent battery design is outlined, alongside open challenges: inversion non‐uniqueness under low‐signal conditions and translation from model cells to practical batteries. This work provides actionable guidelines to leverage NDP for bridging microscopic lithium mechanisms and rational design of next‑generation batteries with high safety and high energy density.

## Graphical abstract

Neutron depth profiling exploits the ^6^Li(n,^3^H)^4^He reaction to provide quantitative, isotope‐specific, depth‐resolved maps of lithium inside operating lithium‐ion batteries, directly linking lithium transport, dendrite growth and interfacial short‐circuiting to a physics‐informed machine‐learning roadmap that turns this non‐destructive operando measurement into a basis for intelligent battery design.

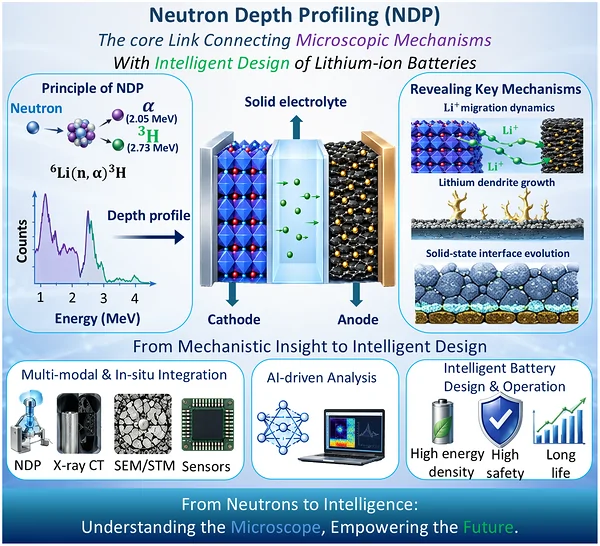

## 1 Introduction

As a key technology for promoting the popularization of electric vehicles and the storage of renewable energy, the performance optimization of lithium‐ion batteries has always converged on the core triangle of energy density, cycle life, and safety. However, the optimization of macroscopic electrochemical performance has reached a plateau [1]. Traditional electrochemical techniques such as cyclic voltammetry (CV) and electrochemical impedance spectroscopy (EIS) provide volume‐averaged, macroscopic information on reaction kinetics, reversibility, and impedance, but they do not directly resolve the real‑time spatial distribution of lithium ions inside the electrode material [2, 3], nor their migration dynamics or the coupling between these dynamics and microstructural evolution [4]. For instance, under low‐temperature or high‐rate charging conditions, lithium ions cannot be intercalated into the graphite anode in time but instead plate on the surface or interior as metallic lithium, forming lithium dendrites. Dendrites not only consume active lithium and cause capacity decay, but may also puncture the separator and cause internal short circuits, which are one of the main causes of thermal runaway. To understand and suppress such failure modes, a tool that can “see” [5] the underlying lithium dynamics inside the battery is urgently needed.

Addressing these diagnostic challenges requires a characterization approach that can non‐invasively bypass battery encapsulation to interrogate light elements with high precision. In this context, Neutron Depth Profiling (NDP) has emerged as a powerful technique for interrogating these internal processes. As a non‐destructive, quantitative, and in situ analysis technique grounded in neutron‐induced nuclear reactions, NDP leverages the high cross‐section nuclear reaction between thermal neutrons and ^6^Li isotopes to decode the depth distribution information of lithium elements inside materials by measuring the energy loss of charged particles generated by the reaction [6, 7]. NDP derives its unique efficacy from a synergy of isotopic specificity, exceptional penetration through battery casing, and high sensitivity [8], thereby enabling precise capture of lithium concentration gradients within electrodes and of the accumulation or depletion of lithium at interfaces (i.e., the local build‐up or removal of lithium at the electrode/electrolyte boundary).

In recent years, major global neutron source facilities (such as NIST in the United States, FRM‐II in Germany, and CARR in China) have continuously sophisticated their NDP spectrometers, significantly improving neutron flux and detection efficiency, making it possible to track the dynamic process of lithium migration during battery cycling in real time [9, 10]. At the same time, data analysis methods have undergone a transformation from traditional linear inversion to intelligent algorithm‐driven methods. A particle swarm optimization maximum likelihood expectation maximization (PSO‐MLEM) hybrid algorithm, first introduced for neutron spectrum unfolding [11], has been adapted to NDP inversion, which effectively solves the ill‐conditioned problems and local optimum traps in the inversion process, improving the accuracy and reliability of data analysis.

The application of NDP has expanded from early material characterization to the core frontier of lithium‐ion battery research. It has not only quantitatively revealed the preferential migration path and significant volumetric strain of lithium in alloy anodes (such as Sn and Si), but also made notable discoveries regarding the growth mechanism of lithium dendrites within solid electrolytes (rather than penetrating from the anode) [12], and the peculiar “reversible short circuit” phenomenon in garnet electrolytes [13]. These findings have not only deepened the fundamental understanding of lithium battery failure mechanisms but also directly guided the design of novel electrode structures (such as gradient electrodes) and solid electrolytes.

Given that current battery research is moving toward multi‐scale and data‐driven directions [14, 15], the role of NDP is increasingly being positioned, together with complementary measurements, as one component of emerging multimodal, data‐driven battery‐analysis frameworks, although this integration remains at an early, conceptual stage. The central scientific question addressed in this review concerns how to quantitatively determine depth‐resolved lithium distribution, isotopic origin, and associated electrochemical consequences within operating batteries—a challenge that can be uniquely tackled by NDP. More specifically, stemming from its distinctive capability, it provides quantitative, isotope‐specific (^6^Li), depth‐resolved lithium information that no other technique can readily deliver. Concretely, NDP addresses three facets of this question: (1) how much lithium is present at each depth, quantitatively and traceably, by exploiting the well‐defined ^6^Li(n,^3^H)^4^He reaction and its 940‑barn cross‑section; (2) which lithium, and from which source, by means of ^6^Li/^7^Li labeling, an isotopic discrimination inaccessible to electron‑ or x‑ray‑based probes; and (3) with what consequence, by locating where and when this quantitatively resolved lithium becomes electrochemically inactive or forms a conductive shorting path, thereby providing a direct basis for linking the measured lithium profile to capacity fade and safety [16, 17]. In moving from “where is the lithium” to “how much, of which origin, and with what consequence”, NDP supplies the quantitative, isotope‐resolved evidence required to advance battery science from trial‐and‐error optimization toward physics‐based design. Looking forward, the integration of multimodal in situ characterization and artificial intelligence analysis offers a promising pathway to promote battery development from trial‐and‐error iteration toward rational design.

Prior reviews have described the principles of NDP and surveyed its early applications in battery research [18]. This review extends this foundation in three interconnected directions to bridge the gap between qualitative characterization and quantitative, mechanism‐driven understanding. First, it incorporates the latest instrumental advances, particularly position‐sensitive N4DP detection, three‐dimensional tomographic mapping, cold‐neutron metrology, and machine‐learning‐based inversion reported recently [19]. Secondly, a systematic and critical assessment is provided regarding actual capabilities and limitations, including quantitative detection limits, calibration and traceability, the role of isotope abundance in quantification, and the direct comparison with competing depth‐resolved probes, as discussed in the literature [20, 21, 22]. Table 1 provides a side‐by‐side evaluation of NDP against the most relevant operando and depth‐resolved techniques, including operando NMR/MRI, neutron imaging, neutron reflectometry, synchrotron x‐ray tomography, cryo‐EM, SIMS, XPS, neutron diffraction, and electrochemical methods, across metrics such as probed quantity, spatial resolution, isotope selectivity, depth‐profiling capability, operando compatibility, and principal limitations, with key references for each entry provided. The comparison reveals that NDP's distinctive value lies not in any single superior attribute, but in a specific combination: ^6^Li isotope selectivity together with quantitative, non‐destructive, in situ depth profiling, a combination that no other single technique can readily replicate. This comparative framework clarifies where NDP is uniquely informative and where it must be complemented by other methods. Thirdly, most distinctively, the abstract concept of “intelligent battery design” is translated from a general aspiration into a concrete roadmap: a closed measurement–analysis–design loop in which NDP serves as the isotope‐specific, quantitative measurement core and physics‐informed machine learning serves as the analytical engine, with the status of each step, whether already demonstrated or still prospective, explicitly distinguished. Nevertheless, this unique combination of capabilities is accompanied by practical constraints including limited access to high‐flux neutron facilities, spatial resolution trade‐offs and uncertainties associated with reconstruction models, which are elaborated throughout the subsequent sections.

**TABLE 1:** Comparison of NDP with complementary and operando characterization techniques for lithium‐ion battery research.

Technique	Probed quantity	Spatial/depth resolution	^6^Li/^7^Li	Quantitative Depth profiling	Non‐destructive/operando	Main limitations
NDP [23, 24]	Li concentration vs. depth	Depth: ∼50 nm near surface, ∼100–200 nm at 1 µm, micrometre scale at maximum depth; lateral ∼130 µm (pixel detector)	Yes (sensitive only to ^6^Li)	Yes (absolute, traceable)	Yes / operando on encapsulated cells	Probe depth limited (α ≈5 µm; ^3^H ≈30 µm); requires neutron beam facility
Operando NMR/MRI [3]	Li chemical environment, ion dynamics, phase evolution	Bulk / mm‐scale; no depth profile	Partial (^6^Li and ^7^Li are both NMR‐active, but without depth resolution)	No	Yes / operando	Low spatial resolution; no depth information
Neutron imaging [25]	Macroscopic Li/H distribution, electrolyte state	∼10–100 µm	No	Semi‐quantitative	Yes / operando	Lower resolution; no isotope specificity; contrast by attenuation only
Neutron reflectometry (NR) [26]	Depth‐resolved scattering‐length density at planar interfaces	∼1 nm in depth (interface)	No	Model‐dependent (semi‐quantitative)	Yes / operando	Planar thin‐film samples only; no lateral mapping
Synchrotron X‐ray tomography [27]	3D morphology, structure evolution	∼1 µm	No	No	Yes / operando (dose‐limited)	Weak for light elements (Li); radiation dose
Technique	Probed quantity	Spatial/depth resolution	^6^Li/^7^Li	Quantitative Depth profiling	Non‐destructive/operando	Main limitations
Cryo‐EM [28]	Atomic‐scale structure and morphology of beam‐sensitive materials	Atomic	No	No	No (destructive, ex situ)	Destructive; non‐quantitative; not operando
SIMS [29]	Elemental/isotopic depth profile	nm–µm depth; sub‐µm lateral	Yes (^6^Li/^7^Li resolvable)	Semi‐quantitative (matrix effects)	No (sputtering is destructive)	Destructive; vacuum required; matrix effects
XPS [30]	Surface chemistry, oxidation state	∼1–10 nm (surface only)	No	No	No (vacuum/ex situ)	Surface‐limited; cannot probe buried interfaces
Neutron diffraction [31]	Crystal structure, phase composition	Bulk/volume‐averaged	No	No	Yes / operando	No spatial Li profile; no isotope selectivity
Electrochemical methods (CV EIS) [13]	Reaction kinetics, reversibility, impedance	Volume‐averaged (no spatial resolution)	No	No	Yes / operando	Yes / operando

## 2 Principle, Instrumentation and Advanced Inversion Algorithms of Neutron Depth Profiling

### 2.1 In‐Depth Analysis of the Physical Principles

The physical basis of NDP technology is the nuclear reaction where thermal neutrons interact with specific isotopes and induce the release of charged particles (n, charged particle). To intuitively illustrate this core physical process, Figure 1 demonstrates a typical in situ NDP measurement configuration for solid‐state batteries. In this setup, the solid‐state cell is placed within a vacuum chamber and irradiated by a thermal neutron beam.

**FIGURE 1:**
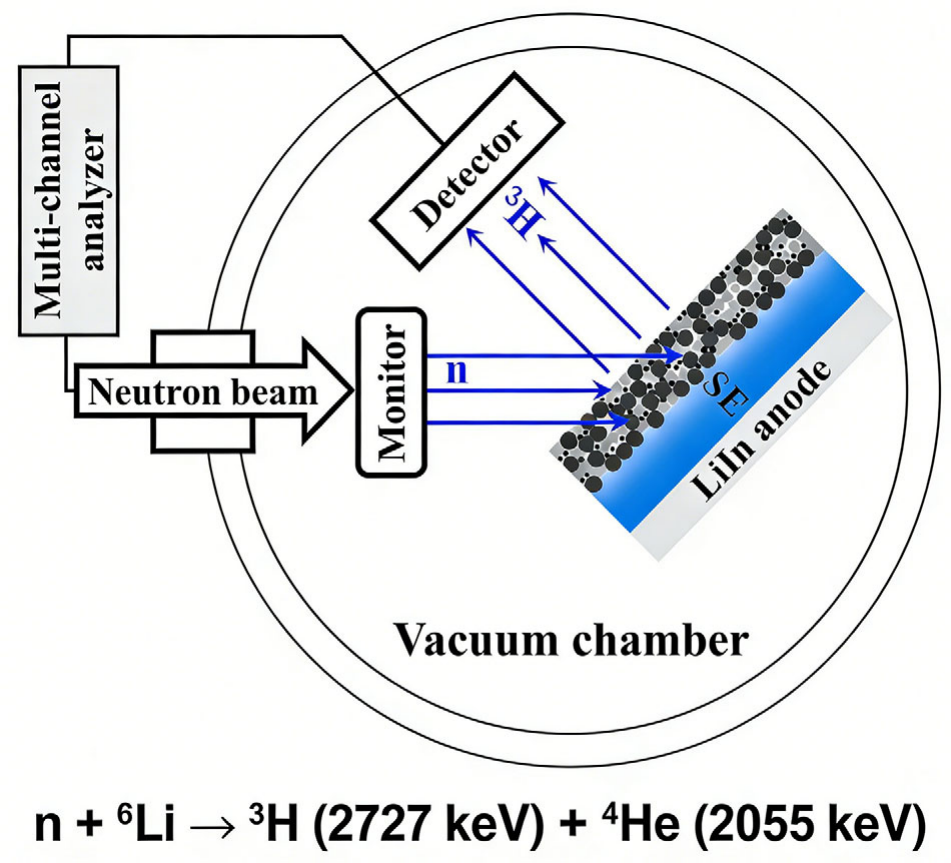
Schematic diagram of NDP measurements for solid‐state batteries.

For lithium‐ion battery research, the core reaction is the thermal neutron capture reaction with ^6^Li:

(1)

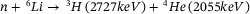




This reaction has a huge thermal neutron cross‐section of 940 barns, facilitating high detection sensitivity. The tritium (^3^H) and alpha particles (^4^He) produced in the reaction are emitted in opposite directions at an angle of approximately 180°, carrying a total kinetic energy (Q‐value) of approximately 4780 keV [32]. This high Q‐value and well‐defined initial energy make the emitted particles readily distinct, serving as a precise reference energy for subsequent depth profiling. As these charged particles traverse the material, they undergo continuous energy loss through electronic interactions, thereby encoding the depth information of the originating reaction site into the measured residual energy.

In addition to ^6^Li, NDP also has a thermal neutron capture cross section of up to 3840 barns for 10B. The latest research has achieved high‐precision quantification of boron implantation dose in SiC using the core reaction in the newly built cold neutron NDP system at the Korea Atomic Energy Research Institute (KAERI) [29]. The combined uncertainty is as low as 1.84%, and the metrological reliability of NDP in near‐surface light element quantification has been confirmed by cross‐validation with secondary ion mass spectrometry (SIMS). This work further consolidates the methodological basis of NDP as an isotope‐specific, traceable absolute quantification technique.

The hallmark of NDP lies in the intrinsic ability of neutrons to distinguish lighter elements and accurately differentiate between isotopes [33]. It is only sensitive to ^6^Li (natural abundance ∼7.5%), while being transparent to ^7^Li. This characteristic makes isotope tracing possible. For example, by using a ^6^Li‐rich electrolyte and a ^6^Li‐depleted cathode material in a battery, NDP can clearly distinguish whether lithium from the cathode or the electrolyte is consumed or deposited during cycling, thus accurately quantifying the migration paths and final distribution of lithium from different sources. Such isotopic resolution is inherently inaccessible to traditional characterization techniques relying on electronic interactions, such as Energy Dispersive Spectroscopy (EDS).

### 2.2 Stopping Power and Depth‐Energy Conversion: The Physical Limits of Resolution

When charged particles (^3^H or ^4^He) traverse from the reaction point to the sample surface, they continuously lose energy through inelastic collisions with electrons in the material. The relationship between the emitted energy *E_det_* and the reaction depth x is governed by the following integral:

(2) $$x=\int_{E_{det}}^{E_{0}}\frac{dE}{S\left(E\right)}$$

where *E*_0_ denotes the initial energy of the particle (e.g., 2727 keV for ^3^H), while *S*(*E*) represents the mass stopping power of the material for charged particles, in units of keV·cm^2^/mg. It is a function of the material composition and particle energy. For the stopping power of composite materials, the Bragg additivity rule is usually used for calculation:

(3) $$S\left(E\right)=\sum_{i}w_{i}S_{i}\left(E\right)$$

Here *w_i_* and *S_i_* (*E*) signify the weight fraction and mass stopping power of each component i, respectively.

The depth resolution of NDP is not constant and can be approximately estimated by the following formula:

(4) $$\delta_{X}\approx \frac{\delta E_{total}}{S\left(E\right)}$$

In this expression: δ*E_total_* accounts for the total energy broadening, which primarily stems from:

1. Intrinsic resolution of the detector: Silicon barrier detectors are typically used, with a resolution of 10–15 keV.
2. Energy straggling: This is the fundamental physical factor limiting resolution, stemming from fluctuations in energy loss due to statistical collisions between charged particles and electrons in the material. Its effect increases with depth.
3. Geometric broadening: Caused by the finite solid angle of the detector, resulting in different path lengths for particles emitted at different angles.
4. Multiple scattering effects: Particles may undergo small‐angle scattering, deviating from their initial path, leading to distortion of energy and depth information.

Consequently, the accumulation of stochastic collision events inevitably leads to the broadening of the energy spectrum as the detection depth increases. In this light, the depth resolution of NDP is not a static parameter but rather a dynamic function of the probing depth, the detected particle species, and the sample matrix. For typical lithium‐ion battery materials (such as graphite and oxide cathodes), the near‐surface depth resolution of NDP is of the order of tens of nanometres (∼50 nm), degrading to ∼100–200 nm at 1 µm depth and to the micrometre scale at the maximum probing depth. This is consistent with the silicon‐detector energy resolution of ∼10–15 keV, which — together with energy straggling, geometric broadening, and multiple scattering — sets the achievable depth resolution [34]. The latest research systematically defined the actual resolution boundary for detection based on alpha particles (2050 keV) and tritium nuclei (2730 keV) [35]: for alpha particles, the effective detection depth is about 5µm, the near‐surface resolution can reach 50 nm, and it broadens to about 100–200 nm at a depth of 1µm; for tritium nuclei, the detection depth can be extended to 30 µm, but the depth resolution is correspondingly reduced to the sub‐micron to micron level. The study also clarifies that for interface layers with a thickness of <100 nm and composed of low atomic number elements (such as Li_2_O), NDP is difficult to form identifiable characteristic signals; the quantitative definition of this resolution limit establishes a rigorous physical benchmark for assessing the viability of NDP in probing buried interfaces within solid‐state batteries.

### 2.3 Overview of Global NDP Devices

The implementation of NDP technology is predicated upon powerful neutron sources and sophisticated detection systems. Several NDP devices have been built worldwide, each with divergent performance parameters and specialties (as shown in Table 2) [36, 37, 38, 39, 40, 41, 42, 43], collectively driving the development of this technology.

**TABLE 2:** Comparison of technical parameters of mainstream NDP devices worldwide.

Facility Name (Country)	Neutron Flux (n cm^−2^s^−1^)	Beam Spot Size (mm)	Detector System Characteristics	Key Advantages and Application Focus
NIST (USA) [38, 43]	1.2 × 10^10^	10 × 10	Multiple silicon detectors, adjustable angle, simultaneous measurement possible.	World's highest steady‐state flux, fast measurement speed, high precision, suitable for high‐precision quantitative analysis and kinetic studies.
FRM‐II (Germany) [39]	3 × 10^9^	11 × 16	Dual high‐resolution silicon detectors, α‐resolution up to 11 keV.	High flux combined with high resolution, enabling in situ battery measurements with 10‐second time resolution [40].
CARR (China) [37, 44]	1 × 10^9^	10 × 10	Single silicon detector, automatic six‐sample position switching.	Independent domestic platform, high vacuum, successfully applied to depth profiling analysis of various domestic battery materials [36].
HANARO [41] (South Korea)	1.84 × 10^8^	20 × 20	Single silicon detector, automatic six‐sample position switching.	Good beam uniformity, suitable for samples requiring large beam spot irradiation.
N4DP [45] (Germany)	5 × 10^10^	20 × 30	Double‐sided silicon strip detector providing simultaneous particle‐energy and impact‐position information; supports two‐ and three‐dimensional imaging.	Position‐sensitive imaging with high detection efficiency; a dedicated light‐element profiling instrument at the Heinz Maier‐Leibnitz Zentrum (MLZ).
CMRR [36] (China)	1 × 10^7^	10 × 10	Single silicon detector, automatic sample switching	NDP facility developed at the China Mianyang Research Reactor; applied to depth profiling of domestic battery materials.

It should be noted that a higher neutron flux does not by itself guarantee better detection efficiency or accuracy. The achievable signal‐to‐noise ratio also depends on the beam‐related background, which includes fast neutrons, gamma rays, and scattered thermal neutrons, as well as on the detector solid angle and efficiency and on the neutron‐scattering cross‐section of the sample. The feasibility of operando or time‐resolved measurements at a given facility should therefore be evaluated not by the nominal flux alone, but by the net lithium count rate achievable per unit time, that is, the useful signal rate after subtraction of the background, and by the counting statistics required to resolve the lithium changes of interest within the desired time window.

The one‐dimensional depth distribution information provided by traditional NDP technology is being significantly augmented through multidimensional imaging techniques [46]. Two‐dimensional lateral imaging, achieved by employing position‐sensitive pixel detectors (as shown in Figure 2a,b) and exploiting the back‐to‐back collinear emission characteristics of ^3^H and ^4^He particles for triangulation, enables the visualization of ^6^Li distribution within the sample plane (as shown in Figure 2c), with a lateral resolution of approximately 130 µm. Simultaneously, three‐dimensional distribution reconstruction techniques, by combining NDP with two‐dimensional mechanical scanning [47], collect depth spectra point by point and superimpose the data, successfully visualizing the three‐dimensional distribution of lithium concentration within a millimeter‐scale volume. This capability proves instrumental in unraveling complex spatial heterogeneities, such as concentration gradients and pore deposition within electrode materials.

**FIGURE 2:**
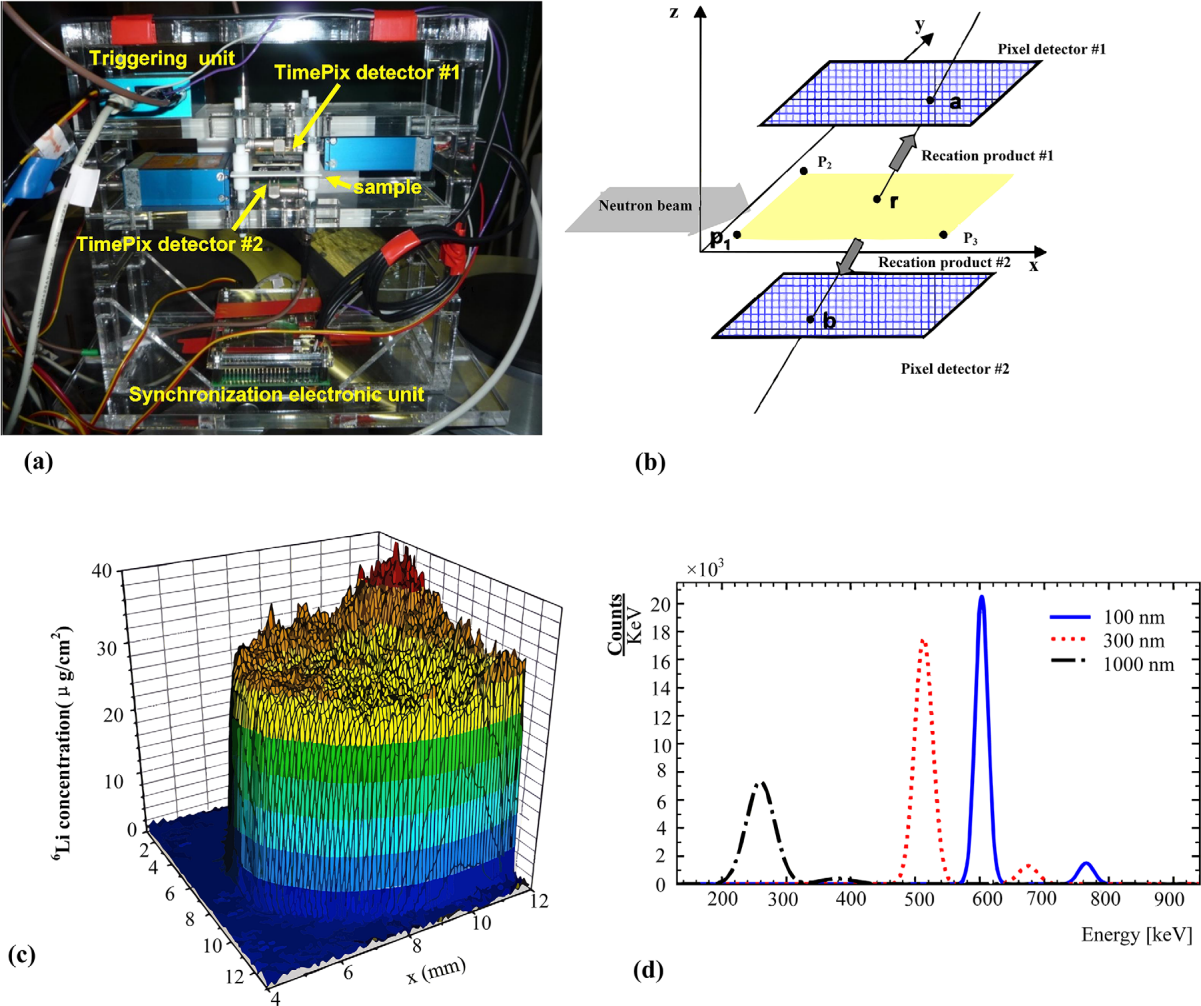
(a): Setup of the position‐sensitive pixel detector (sample placed between two detectors), (b): Schematic diagram of the two‐dimensional imaging principle based on the position‐sensitive pixel detector [46], reproduced from Rev. Sci. Instrum. 88, 023706 (2017), with the permission of AIP Publishing. (c): Two‐dimensional distribution image of ^6^Li in the thin‐layer sample obtained through scientific measurement, (d): Simulation of lithium ion energy resolution under different dead‐layer thicknesses demonstrates how the energy resolution of silicon strip detectors is affected by the dead‐layer thickness, which serves as a critical basis for evaluating detector performance and thereby achieving high‐resolution imaging [45]. Reprinted from Nucl. Instrum. Methods Phys. Res. A 911, 30 (2018), with permission from Elsevier.

The development of next‐generation NDP spectrometers focuses on breakthroughs in imaging efficiency and application range, exemplified by the N4DP concept. The core of this concept is the use of a double‐sided silicon strip detector, which can simultaneously acquire particle energy and impact position information, thus supporting more efficient in situ two‐ and three‐dimensional imaging [45]. Even more forward‐looking is the simulation‐based optimization of dead‐layer thickness in silicon detectors for NDP (as shown in Figure 2d), aiming to mitigate peak broadening and energy shift to achieve high‐resolution analysis. This marks the evolution of NDP from a dedicated lithium analysis tool to a comprehensive bulk element analysis system [48].

### 2.4 Advanced Inversion Algorithms: From Mathematical Solutions to Artificial Intelligence Analysis

The core challenge in NDP lies in accurately reconstructing the measured charged particle energy spectrum *Y*(*E*) to obtain the concentration depth distribution *C*(*x*) of the target nuclide (such as ^6^Li). This process requires solving a system of linear equations.

(5) $$R\cdot C\;\left(x\right)=Y\left(E\right)$$

In this equation, *R* represents the response matrix, typically derived from Monte Carlo simulations (e.g., Geant4), which is inherently ill‐conditioned and renders the inversion process highly sensitive to experimental noise.

Traditional inversion algorithms (such as Singular Value Decomposition Least Squares) face inherent limitations such as ill‐conditioning and noise, easily leading to oscillations or over‐smoothing problems [49]. Contemporary algorithmic advancements represent a transition from deterministic, physics‐based solvers toward probabilistic frameworks incorporating prior knowledge (such as Bayesian estimation), and toward probabilistic frameworks and data‐driven intelligent architectures. This technological chain, from physical fundamentals to intelligent analysis, significantly improves the accuracy and stability of the inversion. Table 3 shows a comprehensive comparison of four inversion algorithms in NDP depth profiling.

**TABLE 3:** Comprehensive Comparison of Four Inversion Algorithms in Neutron Depth Profiling.

Comparison Dimension	Monte Carlo Simulation (e.g.SRIM/TRIM) [50, 51]	Bayesian Estimation [49]	PSO‐MLEM Hybrid Algorithm [11]	Artificial Neural Network [52]
Core Idea / Principle	Uses random sampling to simulate particle transport in complex materials, calculating energy loss, scattering, and other physical effects.	Based on Bayes' theorem, combines observed data with prior knowledge of the concentration distribution to obtain a posterior probability distribution.	Integrates the global search capability of Particle Swarm Optimization with the fast convergence of the Maximum Likelihood Expectation Maximization algorithm, guiding the particle swarm with MLEM iterations.	Constructs a multi‐layer network model to directly learn the complex, nonlinear end‐to‐end mapping relationship between raw spectra and depth concentration profiles.
Main Advantages	1. High Physical Fidelity:Accurately models key physical effects. 2. Flexibility:The gold standard for constructing response matrices and calibration curves. 3. Foundational: Provides the “forward model” for all other algorithms.	1. Incorporates Priors, Robust Results: Effectively suppresses noise‐induced oscillations. 2. Provides Uncertainty Quantification: Outputs a probability distribution of solutions. 3. Strong Capability with Low SNR Data.	1. Excellent Global Convergence: Avoids local optima traps. 2. Fast Convergence Speed: Significantly reduces the number of iterations required. 3. High Inversion Accuracy: Demonstrated the most stable and reliable performance in tests.	1. End‐to‐End Automation: Avoids tedious manual processes. 2. Strong Capability with High‐Dimensional, Noisy Data. 3. Extremely Fast Inference: Predictions are nearly instantaneous. 4. High Accuracy: Predictions show excellent agreement with ground truth.
Limitations /Challenges	1. High Computational Cost. 2. Relies on Accurate Material Parameters. 3. Primarily a forward simulation tool; must be used in conjunction with other inversion algorithms.	1. Results Influenced by Prior Choice. 2. Computing the posterior distribution can be complex.	1. Algorithm Parameters Require Tuning. 2. Slightly less automated compared to pure ANN approaches.	1. Requires Large Amounts of High‐Quality Training Data. 2. Poor Model Interpretability (“black box”).

Comparison Dimension	Monte Carlo Simulation (e.g., SRIM/TRIM) [50, 51]	Bayesian Estimation [49]	PSO‐MLEM Hybrid Algorithm [11]	Artificial Neural Network [52]
Typical Application & Performance	Used to generate the response matrix and calibration function Forms the basis for Bayesian inversion and generating ANN training data.	Applied by Danilov et al. [50] to invert Li depth profiles in all‐solid‐state thin‐film batteries, using the derivative of the calibration function as a prior constraint to obtain more physically reasonable profiles.	Tests by Li Yuanhui et al. [43] show that its correlation coefficient and relative average error are significantly better than or equivalent to those of MLEM, basic PSO, and SVDLS algorithms.	1. Concentration Prediction: Hossny et al. [53] predicted B concentration with R‐scores of 0.94‐0.99. Albarqi et al. [54] proved its predictions are more accurate than traditional methods. 2. Data Preprocessing: Used for Pulse Shape Discrimination to improve SNR.
Role in the NDP Analysis Workflow	Cornerstone of Physical Modeling. Provides an accurate physical mapping from depth to spectrum, bridging the gap between physical principles and mathematical inversion.	Framework for Probabilistic Inversion. Regularizes ill‐posed problems by incorporating prior knowledge, yielding more reliable and interpretable solutions.	Intelligent Optimization Solver. Provides an efficient and robust numerical optimization scheme for complex, non‐convex inversion problems, suitable for high‐precision quantitative analysis.	Data‐driven engine (emerging). Represents a prospective direction for automated analysis; explored for core inversion, data denoising, and feature extraction, and identified in this review as a key component of the proposed multimodal, intelligent‐design loop (Chapter 4).

At the level of intelligent analysis, the latest research introduces artificial neural networks (ANN) into the pulse shape discrimination (PSD) task of NDP [52] to solve the problem that low‐energy background signals seriously interfere with the accuracy of analysis in solid electrolyte measurement. This study enhances the ability of the model to identify low‐energy signals by injecting white noise into the training data. Compared with the traditional rise time method, the ROC(Receiver Operating Characteristic) curve area of the developed ANN‐PSD algorithm is increased by 77%, which significantly improves the signal discrimination efficiency under low signal‐to‐noise ratio conditions. More importantly, the research team introduced interpretable artificial intelligence (XAI) technology to visualize the decision boundary of the algorithm and established a technical chain of “black box model → interpretable features → physical meaning backtracking” in NDP data analysis, offering a robust methodological blueprint for the trustworthy deployment of deep learning within the domain of nuclear spectroscopy.

The practical performance of an NDP measurement is governed by several interdependent parameters that must be carefully considered for reliable quantification. Depth resolution degrades with increasing probing depth: near the surface it is of the order of tens of nanometres (∼50 nm), broadening to ∼100–200 nm at 1 µm and reaching the micrometre scale at the maximum probing depth; the 5–10 nm near‐surface value reported for the cold‐neutron NIST system is only achievable under its specific conditions, while lateral resolution is determined by the neutron beam spot and detector pixel size, typically ∼130 µm for position‐sensitive detectors [24, 34, 46]. The detector–sample geometry, particularly the finite solid angle, introduces geometric broadening because particles emitted at different angles traverse different path lengths, adding to the energy spread. Background signals arise from fast neutrons, gamma rays, and scattered thermal neutrons; effective suppression relies on collimation, shielding, pulse‐shape discrimination, and careful background subtraction. Porous, multiphase, and non‑uniform electrodes violate the ideal uniform‑thin‑layer assumption, causing the measured profile to be smeared; the Bragg‑additivity stopping‑power inputs must therefore be adjusted to the local composition. Operando compatibility demands low‑atomic‑number windows and casings (e.g., Kapton or Al foil) that minimise neutron scattering while preserving electrochemical function. The inversion of the measured spectrum to obtain a depth profile is inherently ill‑posed, leading to non‑unique solutions at low signal‑to‑noise ratios and sensitivity to the assumed response matrix and stopping‑power data [55]. Finally, interpretation of lithium profiles in complex battery electrodes should always be complemented by structural and chemical information from other techniques, since different spatial distributions can produce similar energy spectra. These parameters collectively set the boundaries of what NDP can reliably resolve and should be reported explicitly to ensure reproducibility across studies.

### 2.5 Quantitative Foundations: Detection Limits, Calibration, and Isotope Abundance

The quantitative interpretation of NDP data rests on three considerations that must be made explicit. The first is that of detection limits, since sensitivity is determined by the neutron flux, the reaction cross‐section, and the background level [56]. For lithium, reported near‐surface detection limits are of the order of ∼10^15^ atoms cm^−2^ (roughly a sub‐monolayer to monolayer equivalent), and can be improved with higher neutron flux and lower background. A higher flux alone does not guarantee better sensitivity, however, if the background scales with the flux [57]. Calibration and traceability constitute the second requirement [58], because absolute quantification is achieved by referencing the measured energy spectrum to the reaction kinematics and to the stopping power of the sample matrix, which gives NDP a traceable, calibration‐free character (relative to internal standards) for light elements. Cross‐validation against secondary‐ion mass spectrometry (SIMS) has demonstrated agreement at the ∼1.8% level for boron quantification. Isotope abundance forms the third, since NDP is sensitive only to the ^6^Li isotope (natural abundance ∼7.5%) and the conversion of the measured ^6^Li signal into a total lithium concentration therefore assumes a known ^6^Li fraction. Isotope‐labelled experiments deliberately modify this abundance, which is a powerful feature for tracing lithium originating from different sources, but also requires the isotope ratio to be precisely characterised for absolute quantification to be valid.

## 3 Applications of NDP in Lithium‐Ion Battery Research

NDP technology has successfully aligned the cognitive gap between macroscopic electrochemical performance and microscopic ionic behavior, thereby furnishing a depth‐resolved (nanometre‐to‐micrometre) perspective on the evolution of the lithium distribution, essential for understanding battery operation, optimizing material design, and diagnosing failure modes. Figure 3 illustrates the four core application areas of NDP in lithium‐ion battery research: electrode material kinetics, lithium dendrite behavior, solid‐state battery interface science, and the influence of external conditions, along with specific research examples.

**FIGURE 3:**
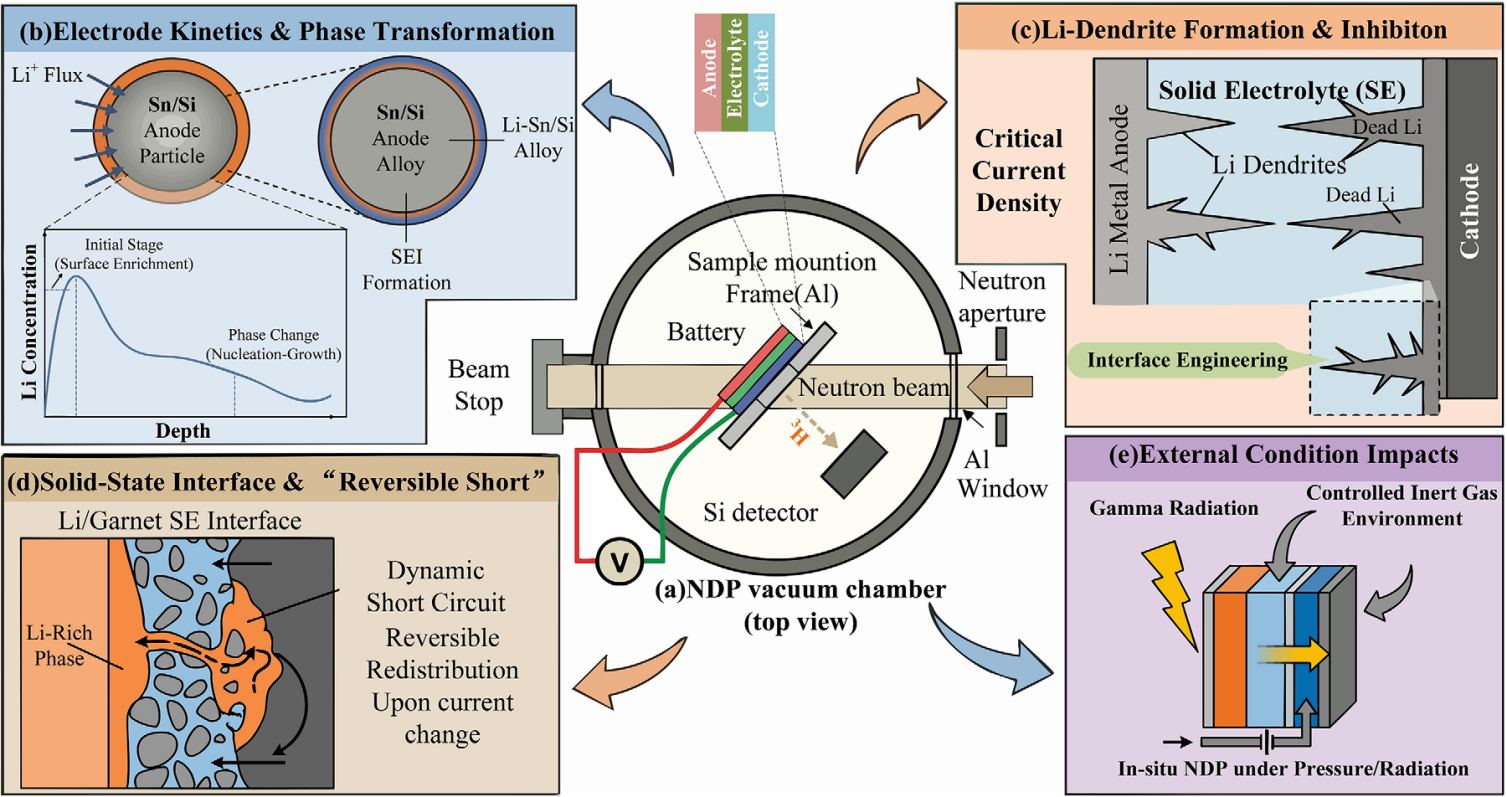
Multi‐dimensional in situ characterization of lithium‐ion battery mechanisms via Neutron Depth Profiling (NDP). (a): NDP System Configuration, (b): Electrode Kinetics & Phase Transformation, (c):Li‐Dendrite Formation & Inhibiton, (d):Solid‐State Interface & “Reversible Short”, (e): External Condition Impacts.

### 3.1 Unveiling the Lithium‐Ion Transport Kinetics and Phase Transition Mechanisms in Electrode Materials

Among alloy‐type anodes, Sn and Al have attracted sustained interest because of their high theoretical capacities. However, both materials undergo drastic volume expansion upon lithiation, often exceeding 300%, which leads to severe mechanical degradation and poor cycling stability. Revealing how lithium infiltrates through these electrodes and how such processes couple to volume evolution is therefore critical to the development of durable alloy anodes. In this context, NDP has emerged as a proven technique for directly probing lithium depth distribution and electrode swelling. As shown in Figure 4a–d, Figure 4a,b presents representative NDP studies of Sn anodes, whereas Figure 4c,d shows the corresponding behavior of Al anodes.

**FIGURE 4:**
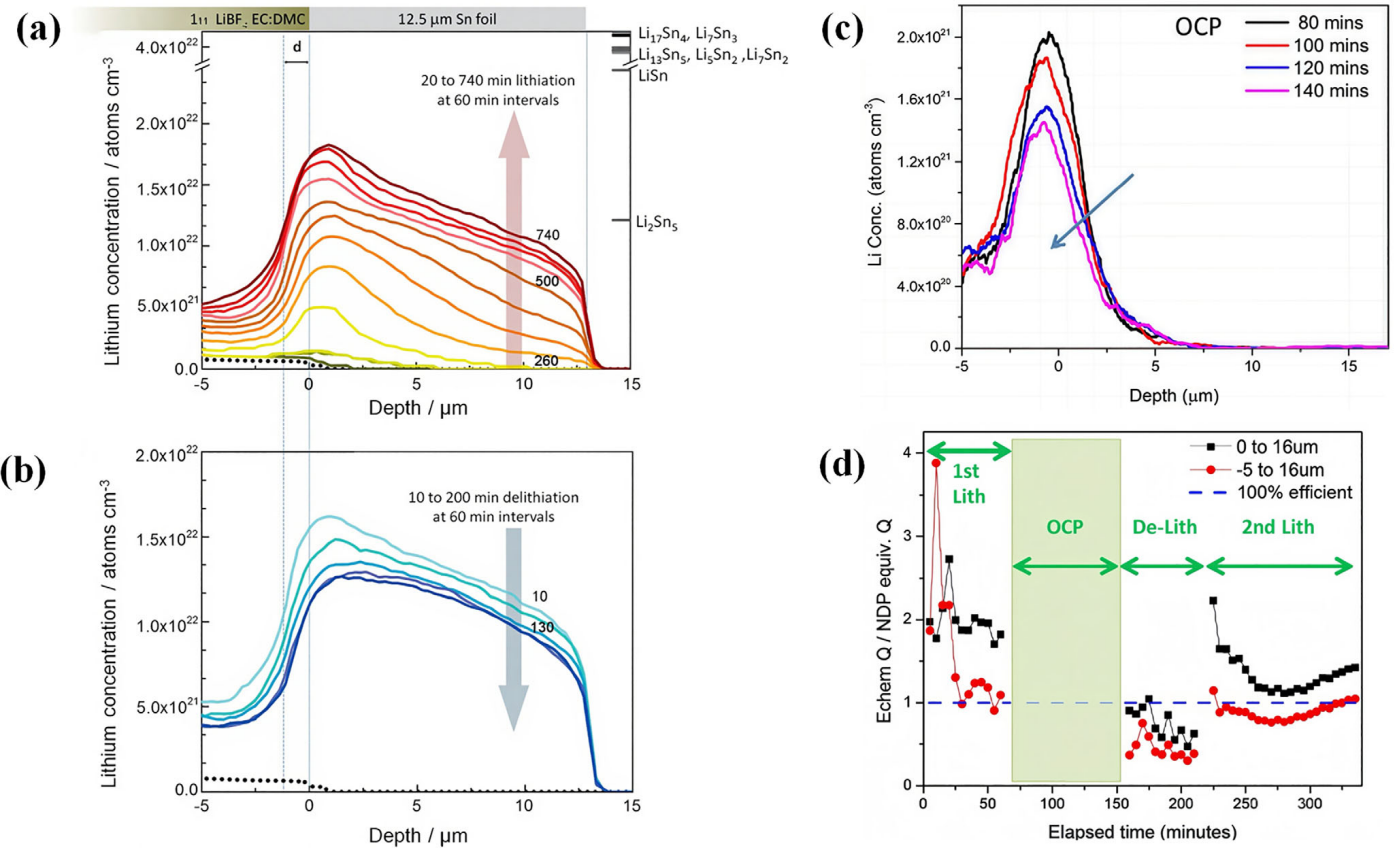
(a): Lithium concentration depth profiles during the lithiation of Sn anodes, (b): Lithium concentration depth profiles during the delithiation of Sn anodes [59], Reprinted with permission from Angew. Chem. Int. Ed. 53, 9498 (2014). Copyright 2014 Wiley‐VCH Verlag GmbH & Co. KGaA. (c): Evolution of lithium concentration under open‐circuit potential (OCP) for Al anodes (revealing self‐discharge behavior), (d): Evolution of the ratio of electrochemical charge to NDP equivalent charge for Al anodes (for quantitative analysis of parasitic reactions) [60]. Reprinted with permission from J. Am. Chem. Soc. 138, 231 (2016). Copyright 2016 American Chemical Society.

In the case of Sn electrodes, the development of an in situ NDP cell tailored for liquid electrolytes, facilitated real‐time, time‐lapse visualization of lithium transport kinetics during Sn electrode lithiation with micrometre spatial resolution and continuous operando acquisition [59]. The study revealed a two‐stage lithiation mechanism: in the initial stage, lithium significantly accumulates in the near‐surface region (0–2µm) of the electrode, reaching a concentration of 1.9 × 10^22^ atoms/cm^3^ before diffusing into the bulk. By establishing a quantitative model between electrochemical charge and the number of lithium atoms detected by NDP, the temporal distinction between effective lithiation and side reactions (such as SEI formation and Sn oxide reduction) was achieved, revealing that approximately 50% of the charge was consumed by side reactions during the first 230 minutes of charging. NDP‐derived concentration profiles also enabled estimation of the apparent (Fickian) diffusion coefficient of lithium in Sn, which decreased from ∼1.5 × 10^−7^ cm^2^/s to ∼0.7 × 10^−7^ cm^2^/s as lithiation proceeded. This value represents an effective, concentration‐averaged diffusion coefficient under the assumption of Fickian diffusion, while concentration‐dependent or two‐phase transport would require more detailed modelling [61].

For Al electrodes: relevant research used in situ NDP to track the distribution of lithium atoms during the lithiation/delithiation process of aluminum anodes at the micrometre scale, revealing its unique “self‐discharge” behavior [60]. Specifically, the continuous decrease in lithium concentration under open‐circuit potential (OCP), as highlighted in Figure 4c, provides direct evidence of internal self‐discharge—a phenomenon that remains elusive to traditional voltage‐based diagnostics. By comparing the electrochemically passed charge Q_electrochemical_ with the charge‐equivalent lithium inventory measured by NDP, Q_NDP_ (converted from the total number of detected ^6^Li atoms), the ratio Q_NDP_/Q_electrochemical_ was used to quantify the fraction of the passed charge that produced electrochemically active lithium [62]. A ratio below unity indicates that part of the charge was consumed by parasitic processes (e.g., SEI formation or oxide reduction) rather than by reversible lithiation.

In the study of silicon‐graphite composite anodes, NDP studies overcame the depth‐of‐field and quantitative constraints, inherent to conventional characterization techniques, achieving depth‐resolved lithium profiling with nanometre‐to‐sub‐micrometre depth resolution [63]. The study found that the lithiation of silicon nanoparticles is directly related to the distribution of electrolyte decomposition products, and provided quantitative lithium‐profile evidence associated with the electrolyte‐drying phenomenon induced by electrode expansion. Because NDP directly measures lithium rather than the electrolyte, this drying is inferred indirectly and should be corroborated by complementary (e.g., ultrasonic) measurements. Further systematic NDP experiments showed that even at 20 wt% silicon, the electrode bulk maintained a uniform lithium distribution, revising the view that high‐silicon electrodes are limited by lithium diffusion kinetics [64]. Separately, operando neutron imaging (not NDP) has guided gradient‐electrode design for high‐mass‐loading cathodes [65]; this complementary neutron technique is mentioned here to illustrate the broader landscape of neutron‐based battery characterization and is not an NDP result.

Taken together, the Sn, Al, and silicon–graphite studies illustrate both the common strengths and the system‐specific limits of NDP. Across all three anode chemistries, NDP consistently separates reversible lithiation from side reactions and resolves the spatial distribution of lithium; however, the observed behavior differs by chemistry, with metallic anodes such as Sn and Al exhibiting pronounced near‐surface accumulation and self‐discharge, whereas the silicon–graphite composite maintains a comparatively uniform bulk distribution at 20 wt.% Si. These contrasts caution against transferring quantitative conclusions, including diffusion coefficients and side‐reaction fractions, from one anode system to another.

### 3.2 Formation Mechanisms, Growth Behavior, and Suppression Strategies of Lithium Dendrites

The uncontrolled growth of lithium dendrites is a core challenge limiting the development of high‐energy‐density lithium batteries. Their microscopic, dynamic nature and deep embedding within the electrode/electrolyte interface make direct observation particularly difficult.

Using NDP technology, relevant research has revealed the spatially non‐uniform statistical nature of lithium dendrite nucleation in solid‐state electrolytes [66]: the accumulated lithium is nearly uniform over large areas (>0.3 cm^2^) yet locally heterogeneous. This indicates that nucleation is governed by the local confluence of several factors rather than by defects alone: the local defect/interface structure, the local electronic conductivity (which determines where Li^+^ can be reduced and Li^0^ can nucleate), local current‐density focusing at contact points or pores, and local mechanical stress [67]. NDP maps the resulting lithium distribution as a statistical signature of nucleation, whereas the underlying defect structure itself requires complementary electron‐microscopy characterization [68]. When the NDP detection area exceeds 0.3 cm^2^, the measured lithium distribution is nearly uniform, which contrasts sharply with the non‐uniform morphology observed in local images from scanning electron microscopy, thus confirming that dendrite formation is a statistical phenomenon resulting from the synergistic effect of intrinsic material defects and the external electric field. By quantifying the deviation between the accumulated charge and the actually detected lithium transport amount (Figure 5), the study identified a widening divergence that signifies the onset of an electrochemical side reaction or the spatial sequestration of lithium into electrochemically inactive filaments(dendrites). Consequently, the researcher determined the critical point for the deep formation of lithium dendrites inside electrolytes such as LLZO and Li_3_PS_4_, and established a “charge‐lithium transport efficiency” model, providing a threshold criterion based on electrochemical behavior for predicting dendrite initiation [66].

**FIGURE 5:**
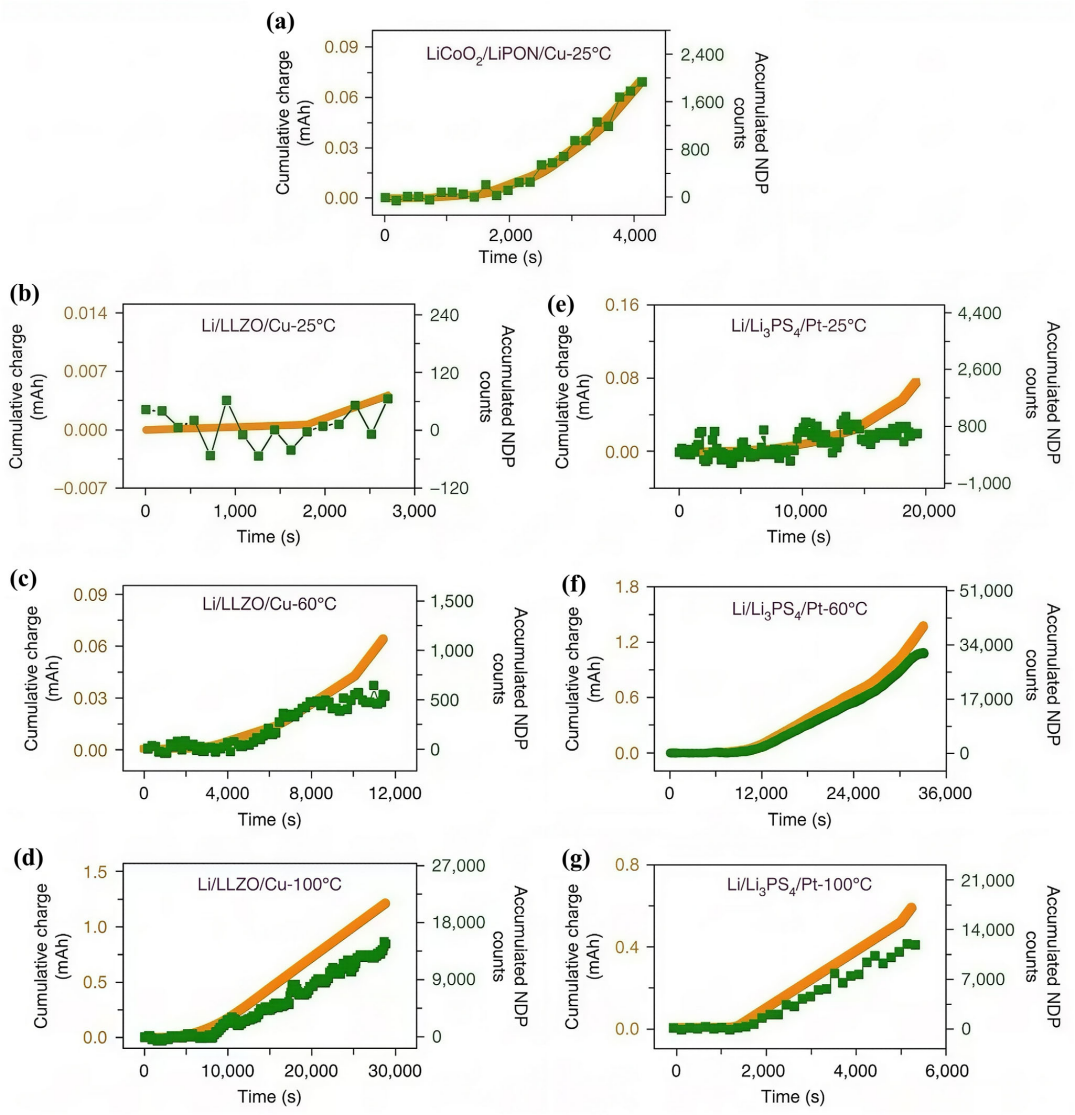
Correlation between cumulative electric charge and NDP‐detected lithium content (Quantifying the deviation for dendrite initiation): Redrawn with permission from Ref. [61]. Correlation between cumulative electric charge and NDP‐detected lithium content (Quantifying the deviation for dendrite initiation): Correlation between electric charge and accumulated Li content. Figure (a–g) illustrate the correlations between the cumulative charges (orange line) and the accumulated NDP counts (green dots) in the total region (surface and bulk) of various cells, specifically corresponding to (a): the LiCoO_2_/LiPON/Cu cell, (b): the Li/LLZO/Cu cell tested at 25°C, (c): the Li/LLZO/Cu cell tested at 60°C, (d): the Li/LLZO/Cu cell tested at 100°C, (e): the Li/Li_3_PS_4_/Pt cell tested at 25°C, (f): the Li/Li_3_PS_4_/Pt cell tested at 60°C, and (g): the Li/Li_3_PS_4_/Pt cell tested at 100°C [66]. Reproduced with permission from Nat. Energy 4, 187 (2019). Copyright 2019, Springer Nature.

In another study, the causal nexus between “dead lithium” accumulation and capacity attrition was quantified by titration gas chromatography (TGC) [69], complemented by cryo‐EM imaging; the bulk dead‐lithium inventory (up to 78.3%±4.2%) was obtained by TGC. NDP's contribution to this problem is its ability to resolve the spatial distribution of the inactive lithium, which is complementary to the bulk TGC quantification. Although increasing the temperature helps to improve ion conductivity, it also accelerates the penetration of dendrites into weak points at the interface (for example, at 60°C, the lithium transport amount increases by about 3.7 times, and the penetration depth increases by 42%). To address this problem, the study proposed a dual‐interface engineering strategy: for example, adding 0.5 wt.% LiNO_3_ can increase the Li_2_O content in the SEI to 73.8%; constructing a Li_3_N buffer layer can significantly reduce the interface energy barrier from 0.78 to 0.31 eV. This strategy enables Li/LLZO/Li symmetric batteries to cycle stably for over 1200 h and increases the critical current density to 1.2 mA cm^−^^2^.

By measuring the lithium concentration gradient at the dendrite front (on the order of 10^2^ ^6^–10^2^ ^8^ atoms cm^−^^4^, corresponding to ∼10^2^ ^2^ atoms cm^−^^3^ varying over a micrometre scale) and its temporal relationship with the electrochemical response, the applicability boundary of the classical “Sand's time” theory has been tested in a practical system [70]; this Sand‐time analysis originates from an optical capillary‐cell experiment, and NDP provides the complementary quantitative lithium‐concentration profiles against which such transport models can be tested. The actual dendrite initiation time was found to be 37%–52% earlier than the classical prediction. This deviation arises from at least three coupled effects that the idealized Sand model neglects: (1) stress‐coupled transport: the mechanical stress generated by lithium plating modifies the local chemical potential and apparent diffusivity, so the transport is no longer purely Fickian; (2) current constriction, arising from interfacial defects and poor contact that focus the local current density and shorten the local Sand time at hotspots; and (3) non‐uniform ion flux, caused by the porous, heterogeneous electrode/electrolyte medium that produces spatially varying ionic flux. NDP provides the evolving lithium concentration profile needed to test these mechanisms, whereas the stress field, current distribution, and flux heterogeneity must be obtained from mechanical modeling or complementary imaging. These findings promote battery design from empirical trial and error to mechanism‐driven approaches, providing a scientific basis for the development of dendrite‐free, high‐energy‐density batteries.

Recently, a study used three‐dimensional tomographic NDP technology to achieve the first quantitative visualization of the spatial distribution of lithium dendrites in solid polymer electrolytes [71]. It was found that the dendrite distribution exhibits significant heterogeneity across the scale of tens of nanometres to centimeters. Strikingly, the high sensitivity of NDP facilitated the observation of counter‐intuitive “reverse growth” from the stripped electrode side—a phenomenon previously obscured in traditional imaging studies.

Comparing these studies, dendrite nucleation is consistently found to be statistically non‐uniform and to precede macroscopic electrochemical signatures, whether in ceramic (LLZO, Li_3_PS_4_), liquid, or solid‐polymer electrolytes; the critical current density, the nucleation‐site distribution, and the degree of spatial heterogeneity, however, are system‐specific, reflecting differences in electronic conductivity and interfacial contact. The cross‐study consistency of the non‐uniform‐nucleation picture, obtained by independent NDP experiments on different electrolytes, is what elevates it from a single‐system observation to a robust mechanistic conclusion.

### 3.3 Interfacial Stability of Solid‐State Batteries and the “Reversible Short Circuit” Phenomenon

The interface stability of solid‐state batteries directly affects battery safety and cycle life, but its atomic‐scale dynamic processes are difficult to observe directly under operating conditions. NDP, with its isotope‐specific identification of lithium and sub‐micrometre depth resolution, has achieved real‐time visualization of lithium transport and short‐circuit mechanisms at the solid‐state electrolyte/electrode interface.

By monitoring the cycling of a Li/Garnet/Li symmetric battery using NDP, the study first identified that “non‐zero counts” in the NDP signal, appearing before significant electrical short‐circuiting, can serve as an early warning signal for short‐circuit risk [13]. This reveals the so‐called “dynamic short‐circuit” mechanism: when lithium is stripped from the top electrode surface, a lithium‐rich phase accumulates at the interface and grows toward the counter electrode, leading to a short circuit; during reverse charging, the lithium in this lithium‐rich phase is stripped back to the original electrode, causing the short‐circuit path to temporarily disappear, forming a transient phenomenon of “reversible short circuit”. This discovery revises the traditional understanding of short‐circuit behavior in solid‐state batteries.

Further research, through systematic NDP experiments, directly confirmed the dynamic mechanism of “reversible short circuit” [72]: poor interface contact at high current densities leads to the preferential formation of a Li‐rich phase at the interface, triggering a short circuit; when the current is reduced or disconnected, the Li‐rich phase redistributes into the garnet matrix, rendering the short‐circuit state partially or completely reversible. Quantitative NDP data show that after short‐circuiting at current densities greater than 0.2 mA cm^−^^2^, the NDP‐measured lithium count deviates from the lithium expected from the passed charge by up to 37% (the “deviation rate”, defined as the relative difference between the accumulated NDP lithium count and the charge‐integral prediction), and when the current is reduced to 0.1 mA cm^−^^2^, the fraction of the short‐circuit‐induced lithium that is recovered, termed the “reversibility”, reaches 82% (Figure 6). This study clarifies that interface contact quality is a core factor in regulating the dynamic evolution of the Li‐rich phase, providing novel paradigms for suppressing short circuits through interface engineering. The study further demonstrates the multidimensional analysis capability of NDP in all‐solid‐state thin‐film lithium batteries. One‐dimensional (1D) NDP resolves the lithium concentration along the depth direction, which is normal to the surface. Two‐dimensional (2D) NDP resolves the lateral distribution in the sample plane together with the depth distribution, with a lateral resolution of approximately 130 µm for position‐sensitive detectors. Three‐dimensional (3D) NDP reconstructs the lithium distribution in both lateral dimensions and in depth by point‐by‐point mechanical scanning. These capabilities show that NDP can monitor the dynamic migration of lithium in solid electrolytes under real‐time operating conditions and can identify lithium depletion and enrichment at interfaces in situ (Figure 7) [73].

**FIGURE 6:**
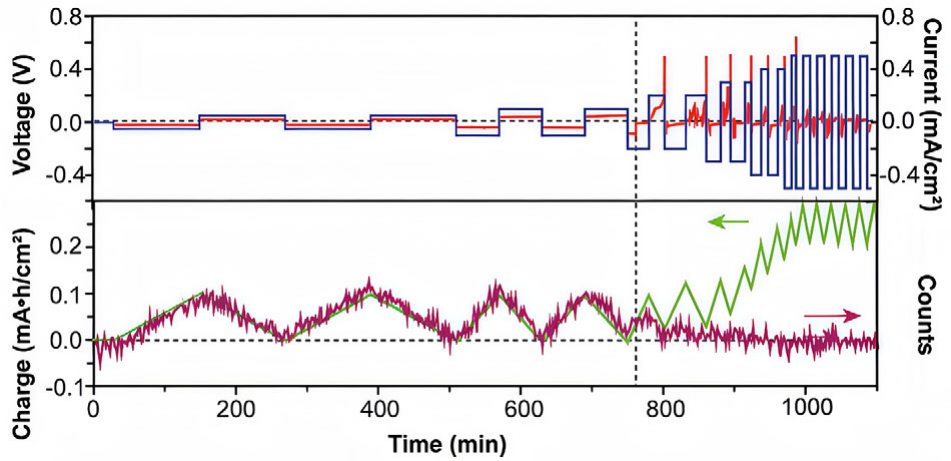
In Situ NDP Reveals Li Transport Behavior During Reversible Short‐Circuit Process [72]. Reprinted with permission from Adv. Energy Mater. 8, 1703614 (2018). Copyright 2018 Wiley‐VCH Verlag GmbH & Co. KGaA.

**FIGURE 7:**
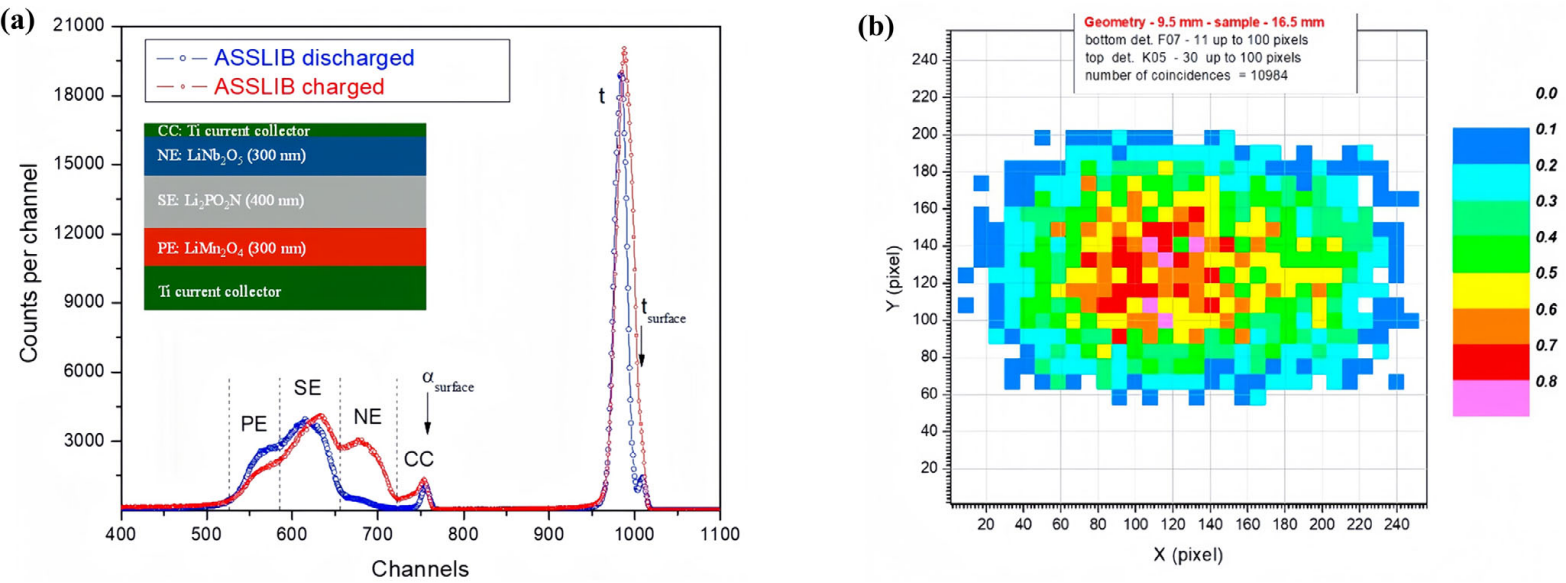
Multi‐dimensional NDP visualization of Li‐ion distribution and migration in ASSLIB: 1D depth profiling(a) and 2D lateral mapping(b) [73]. Reproduced from Radiat. Eff. Defects Solids 179, 1564 (2024), with permission from Taylor & Francis.

NDP precisely quantifies and reveals the microscopic mechanism of “reversible short circuit”: the short‐circuit path is formed by a conductive Li‐rich phase in the ceramic. The microscopic origin of this conductivity can be understood in terms of electronic percolation: isolated lithium precipitates are electronically disconnected, but as lithium accumulates along grain boundaries and defects its volume fraction grows until it exceeds the percolation threshold, at which point a continuous electron‐conducting Li‐rich network spans the electrolyte and shorts the cell [74]. This percolation picture explains two observations. The first is that a “non‐zero” but spatially unconnected lithium signal appears before the actual short, which serves as an early warning. The second is that the short is reversible, because the Li‐rich phase dissolves or redistributes locally when the current is reduced or reversed, and the conductive network is then broken again. The local lithium density measured by NDP can therefore be interpreted as a proxy for how close the system is to the percolation threshold [75]. By in situ monitoring of the interface Li distribution, NDP confirms that optimizing interface contact can suppress the formation of the Li‐rich phase. This discovery promotes solid‐state battery research from a passive strategy of “avoiding short circuits” to an active design of “controllable short circuits”, marking a new stage of mechanism‐driven scientific design in interface engineering.

In addition to directly revealing dynamic failure mechanisms such as “reversible short circuit”, the capability boundary of NDP in solid‐state interface research has also been systematically defined. Some studies have compared the characterization capabilities of NDP and neutron reflection in the buried interface of the Li/LiPON model system, and quantitatively clarified the interface resolution limit of NDP [26]: For a lithium‐rich phase (such as Li_2_O) interface layer, its thickness needs to reach 50–100 nm to be clearly distinguished by NDP; while neutron reflection is highly sensitive to gradient interfaces <30 nm. This study not only emphasizes the strong complementarity of NDP and NR at the 10 nm–1 µm scale, but also points out from a methodological perspective that in solid‐state battery research, a single characterization technique cannot cover all relevant scales. It is necessary to construct a multi‐scale neutron characterization framework such as “NDP (submicron to micron lithium distribution) + NR (nanoscale interface configuration)” in order to fully analyze the whole chain mechanism from interface chemical reaction to device‐level transport behavior.

Across the garnet and thin‐film studies, the reversible‐short‐circuit mechanism is consistent: a Li‐rich phase accumulates, shorts the cell, and redistributes upon current reversal, so the mechanism itself can be regarded as robust. The quantitative values, however (a 37% deviation and 82% reversibility), are specific to the particular garnet cell and cycling protocol. The resolution comparison between NDP and neutron reflectometry at the buried interface further shows that the two techniques are complementary rather than interchangeable, delimiting the scale range over which each is trustworthy.

### 3.4 Impact of External Conditions on Battery Performance

To study the impacts of the external environment, a specialized NDP technique has been developed that operates in a gas‐controlled environment, allowing for in situ measurements of air‐sensitive battery samples in an inert atmosphere (such as argon or helium) [76]. This technique controls the chamber pressure (approximately 1 atm, slightly higher than the electrolyte vapor pressure), enabling in situ observation of liquid electrolyte batteries while maintaining NDP accuracy, providing a new method for exploring the effects of the external gaseous environment.

Another study investigated the effects of gamma radiation on lithium‐ion battery electrolytes [77], quantifying the relationship between radiation dose and battery performance. The study showed that radiation doses up to 2.7 Mrad induce chemical alterations in the electrolyte (discoloration, changes in FTIR characteristics), but the radiation dose used in NDP measurements is only several to tens of Rad, far below the threshold for effects. By establishing that the operational dose of NDP is orders of magnitude below the degradation threshold, this study underscores the non‐perturbing nature of NDP in sensitive electrochemical systems, providing a basis for battery evaluation in extreme environments such as nuclear accidents and space exploration.

Beyond one‐dimensional profiling, the evolution toward three‐dimensional tomographic NDP enables the holistic quantification of lithium dynamics with micron‐level resolution, effectively circumventing the limitations inherent to traditional electrochemical diagnostics. This non‐destructive capability, which avoids the sample damage typical of electron microscopy, is catalyzing the transition from macroscopic performance evaluation to microscopic mechanism elucidation [78]. Future work should prioritize developing multi‐physics coupled in situ NDP techniques to monitor lithium transport dynamics under extreme conditions in real‐time, providing crucial guidance for the design of high‐safety batteries.

These studies address different external perturbations, namely the gas environment, gamma radiation, and three‐dimensional tomography, and therefore do not directly overlap. One conclusion is nevertheless common to all of them. NDP remains non‐perturbing under operando conditions, since its operational dose is orders of magnitude below the degradation threshold. The technique also extends beyond one‐dimensional profiling.

### 3.5 From Model Cells to Practical Batteries

Most of the results above were obtained on model systems, such as thin‐film electrodes, symmetric Li/electrolyte/Li cells, and small‐area pouch cells, because these minimize neutron scattering, simplify the stopping‐power (Bragg) inputs, and keep the lithium signal within the limited probe depth (α ≈5 µm; ^3^H ≈30 µm). Extending NDP to practical architectures introduces several challenges that the field is only beginning to address: (1) for thick, high‐loading electrodes, the NDP probe depth samples only the outermost tens of micrometres, so representative information requires either oriented sampling or multiple spots. (2) Porous‑electrode heterogeneity can distort measured profiles and bias apparent lithium gradients due to non‐uniform spatial distribution of active material, binder, and conductive additive [79]. (3) Testing commercial‑format cells (cylindrical, prismatic, large‑size pouch cells) require low‐atomic‐number windows (Kapton, Al) that preserve electrochemical operation while minimizing background. (4) The small beam footprint relative to a full‐size cell raises questions of statistical representativeness [80]. It should therefore be emphasized that, at present, NDP is quantitatively most reliable in model and thin‐film systems, and that its translation to practical cells should be interpreted with these caveats.

Preliminary efforts including optimized operando cell housing, segmented beam spot scanning and improved Bragg additivity stopping power corrections have been reported to mitigate some of these obstacles, yet complete quantitative translation remains an ongoing open challenge [81, 82]. These practical cell constraints also propagate into the closed loop measurement analysis design paradigm outlined in Section 4. Even with advanced multimodal fusion and physics‐informed machine learning workflows, the intrinsic uncertainties arising from realistic porous electrode architectures must be explicitly accounted for when extending the roadmap from well‐defined model cells toward commercial relevant battery formats.

## 4 Toward NDP‐Enabled Intelligent Battery Design: A Roadmap

To address the deficiencies of traditional single‐point observation, modern lithium‐ion battery research is developing a new paradigm of multimodal in situ characterization and intelligent analysis [83, 84]. As illustrated in Figure 8, the integration of neutron depth profiling, electrochemical calorimetry, and acoustic transmission techniques, coupled with artificial intelligence algorithms, enables synchronous dynamic observation and high‐precision analysis of the internal “lithium distribution–thermal behavior–electrolyte state” evolution in batteries. This provides a cross‐scale, quantitative global research tool for elucidating complex degradation mechanisms and guiding smart battery design.

**FIGURE 8:**
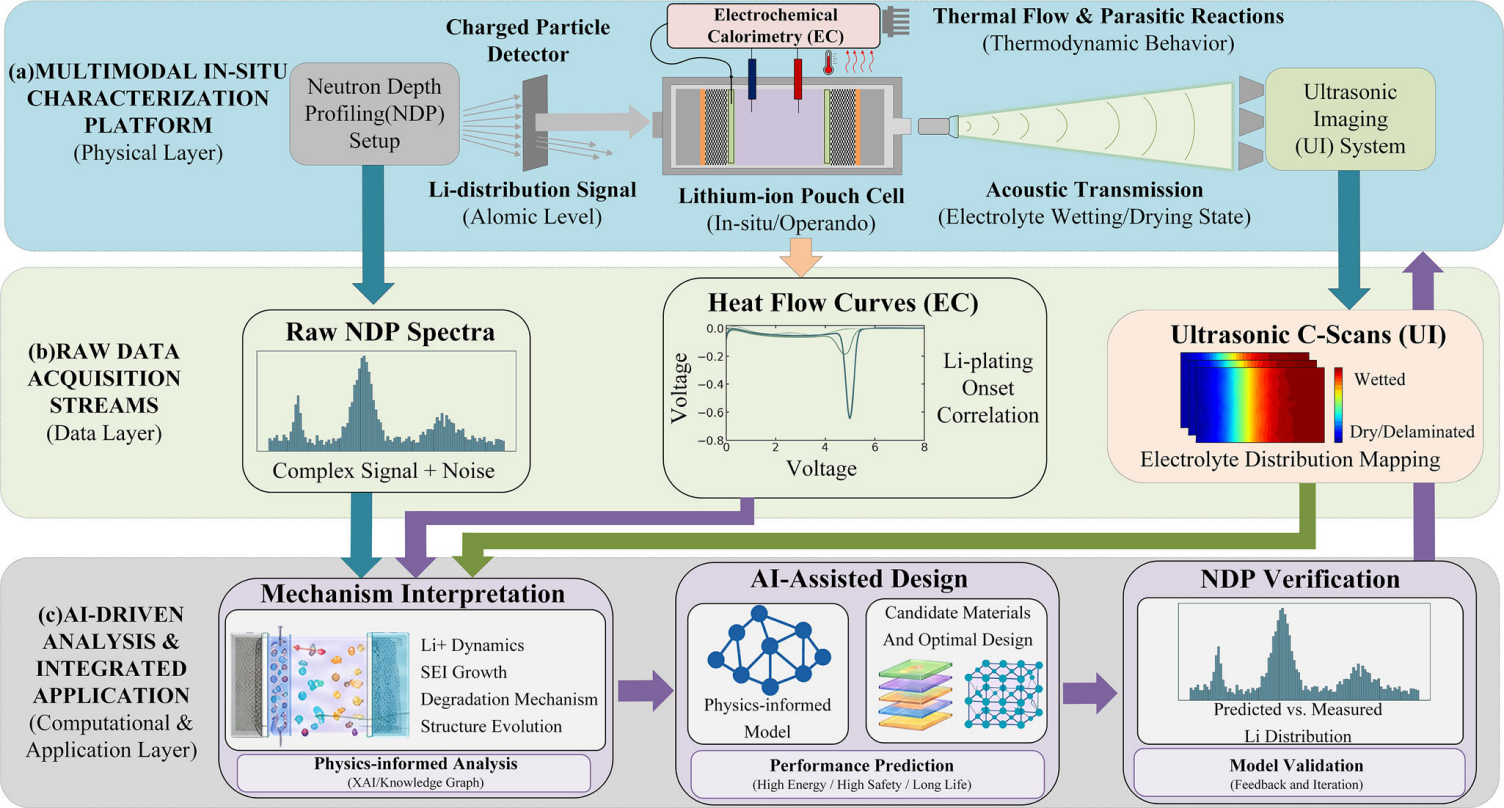
Conceptual roadmap of the proposed closed‐loop, multimodal characterization and AI‐driven analysis framework for next‐generation battery research. The framework is organized as a measurement–analysis–design loop, comprising (a): Multimodal in situ characterization platform (physical layer), (b): Raw data acquisition streams (data layer), (c):AI‐driven analysis & integrated application (computational & application layer).

### 4.1 Multimodal In Situ Characterization: From Separate Demonstrations to a Proposed Integrated Framework

Modern lithium‐ion battery research is evolving toward a collaborative, integrated multimodal in situ characterization platform. A combined NDP–calorimetry–ultrasound platform is a proposed framework rather than an achieved instrument at present. The constituent measurements have each been demonstrated separately, including electrochemical calorimetry for lithium plating, ultrasonic imaging for electrolyte wetting [85], and NDP for lithium distribution. However, their synchronous physical integration has to date only been discussed in a meeting abstract [86] and awaits validation in a peer‐reviewed operando study.

The multimodal platform operates by combining complementary diagnostic modalities to capture distinct battery‑related observables. NDP bombards the battery with thermal neutrons and detects the charged particles produced by nuclear reactions, providing quantitative, depth‐resolved information on the lithium distribution, which is crucial for understanding core mechanisms such as lithium‐ion migration and deposition. Meanwhile, electrochemical calorimetry (EC) simultaneously monitors the battery's heat flow and parasitic reactions, correlating voltage changes with subtle thermal effects, revealing the intensity of side reactions. In parallel, acoustic transmission technology interrogates the mechanical impedance of the cell to non‐destructively characterize the wetting or drying state of the electrolyte, reflecting changes in the physical contact at the electrode‐electrolyte interface.

The advantage of this multi‐technology integration resides in its robust complementarity. For example, electrochemical calorimetry has captured lithium deposition on graphite anodes, but the high degree of overlap between the heat flow drop point and the deposition starting point allowed for quantitative calculation of lithium deposition efficiency (0.98 and 0.97), establishing a quantitative relationship model of “lithium distribution‐thermal response‐electrochemical efficiency” [87]. Furthermore, by combining NDP with ultrasound imaging, dynamic visualization of electrolyte distribution was achieved. It was found that at high temperatures (55°C) and deep cycling (100% DOD), the ultrasound images showed blue regions indicative of electrolyte drying, which was directly correlated with accelerated capacity degradation; while the good wetting state under shallow cycling (25% DOD) corresponded to a lower degradation rate, revealing a direct causal chain between “electrolyte microscopic distribution and macroscopic performance” [85].

The significance of this platform extends far beyond the mere additive accumulation of datasets. It integrates the quantitative lithium‐concentration distribution provided by NDP, the thermodynamic fingerprint of EC, and the mesoscopic structural state of ultrasound, collectively constructing a multi‐dimensional spatiotemporal atlas of “lithium distribution‐thermal behavior‐electrolyte state” from the sub‐micrometre to the mesoscopic scale, providing a multi‐scale, cross‐correlated view of complex battery degradation mechanisms. AI serves as a useful component that transforms disconnected raw signals into a coherent multi‑dimensional atlas. It provides one data‐driven route to solving the inverse problem of extracting lithium distributions from noisy NDP data, complementing Bayesian and optimization‐based (e.g., PSO‐MLEM) inversion methods [88], and algorithmically links them across scales with thermal and ultrasonic fingerprints. This advancement elevates the platform from passive data collection to true mechanistic integration (Figure 8c).

It should be recognized that NDP alone cannot describe the complete state of a battery. NDP provides quantitative, depth‑resolved information on lithium distribution, but thermal, mechanical, structural, and electrochemical states must be addressed by complementary techniques, including calorimetry, ultrasound, x‑ray/scattering, and electrochemistry. Combining these measurements within a coherent physical framework is therefore a necessary step toward a more complete description of battery behavior, rather than a capability that NDP alone can offer.

### 4.2 Raw Data Acquisition Flow: Synchronous Convergence of Multi‐Source Heterogeneous Data Streams

The operation of the multimodal platform yields high‐velocity, multi‐source heterogeneous data streams, including three main categories of core data:

1. Raw NDP spectra: Nuclear reaction signals acquired by the detector are affected not only by background noise, but also by the detector response function, target–detector geometry, and calibration uncertainties. These signals form the basis for inverting lithium distribution, and their signal‐to‐noise ratio influences the inversion accuracy, which also depends on counting statistics, detector resolution, and background‐subtraction procedures [89]. Yet this signal‐to‐noise ratio is not the sole governing factor.
2. Heat flow curves (Electrochemistry‐Calorimetry): High‐time‐resolution voltage and heat flow curves, whose fluctuations correspond to internal battery reactions and phase transitions, used to analyze side reactions and thermal runaway precursors.
3. Acoustic imaging/transmission data: Signals or images acquired by ultrasound, which can dynamically reflect the internal structure and electrolyte wettability gradients of the battery.

The synergy of NDP, electrochemical calorimetry, and ultrasonic imaging mentioned above transcends the mere instrumental concatenation of diagnostic tools. The study also systematically proposed a framework for the deep integration of multimodal data and battery science, and pointed out sharply [90]: no matter how precise a single characterization technique is, it can only capture a discrete projection of the convoluted internal physicochemical field inside the battery, and cannot fully describe its multi‐physical field coupling nature. The real breakthrough lies in the fact that by aligning and co‐modeling the features of heterogeneous data such as NDP (sub‐micrometer‐to‐micrometer lithium distribution), synchrotron X‐ray tomography (mesoscopic structure evolution), ultrasound (macroscopic wetting state), and thermal spectroscopy (reaction thermodynamics), a cross‐dimensional correlation from the atomic scale to the device scale can be established. This constitutes the core methodology of an integrated multimodal monitoring framework, which enables a shift from the physical co‐location of instruments toward a cross‐correlated, model‐based analysis.

The synchronous integration of NDP, calorimetry, and ultrasound is subject to several physical constraints that must be resolved before a combined result can claim greater accuracy than any single modality. Temporal alignment is the first difficulty, because NDP acquisition is governed by neutron count statistics and typically requires seconds to minutes per profile, while calorimetry and ultrasound operate at much higher rates. The modalities must therefore be time‐stamped and interpolated onto a common clock. Spatial registration presents a further problem, since the NDP beam footprint, the calorimetric sensing volume and the ultrasound field of view do not overlap and thus sample different regions of the cell. Calibration introduces a third source of uncertainty, as each modality depends on its own calibration chain, such as stopping power for NDP, thermal calibration for calorimetry, and acoustic coupling for ultrasound. These uncertainties, together with the uncertainty of the NDP deconvolution itself, propagate into the fused estimate. Mutual interference must also be considered, because acoustic transducers, thermal sensors and electrical leads placed near the sample can increase the neutron‐scattering background and perturb the NDP signal. Resolving these constraints is the central technical challenge that must be met before multimodal fusion can be regarded as quantitatively reliable.

### 4.3 From NDP Spectra to Intelligent Battery Design: The Role of Machine Learning

Machine learning (ML), in particular artificial neural networks (ANNs), is beginning to influence NDP data analysis, but its present role should be stated precisely. The methods demonstrated so far are proof‐of‐concept demonstrations, most of them trained on simulated data. AI‐assisted battery design with NDP at its center remains a roadmap rather than an achievement. This section unfolds along three thematic threads. It first elaborates on the inherent suitability of NDP data for machine learning workflows, before summarizing existing experimental demonstrations and outlining a concrete measurement–analysis–design loop that defines NDP‐enabled “intelligent battery design”.

The connection between NDP and machine learning is not coincidental; it arises from the physics of the method. Unlike most battery diagnostics, NDP has a well‐defined linear forward model: the measured energy spectrum Y is related to the lithium concentration profile C by Y = R·C, where the response matrix R can be computed with high fidelity via Monte Carlo simulation of charged‐particle transport, and the depth scale is anchored by stopping power rather than by empirical calibration. Three consequences follow. First, physically realistic training data can be generated at will from the forward model; this addresses the scarcest resource in NDP, namely experimental beam time, and explains why essentially all ANN demonstrations to date have been trained on simulated spectra. Second, the physics can be embedded in the model rather than being replaced by it. An ANN does not eliminate the evaluation of stopping power; the depth scale still rests on the charged‐particle energy loss, while the response matrix can be imposed as a constraint, in the spirit of physics‐informed neural networks. So, the network inherits the correct depth scale instead of re‐learning it from data. This also distinguishes ANN reconstruction from conventional inversion. Singular‐value decomposition, Bayesian, and maximum‐likelihood methods explicitly invert Y = R·C with the physical response matrix and yield interpretable, uncertainty‐quantified solutions. In contrast, a purely data‐driven network learns the inverse mapping end‐to‐end and is fast but poorly interpretable. The most promising route is a hybrid strategy, in which the network retains the physical forward model and learns only the parts that are difficult to model. Third, the ^6^Li‐specific, quantitative nature of the NDP signal gives the learner a clean target, free of the elemental overlaps that complicate most spectroscopic machine‐learning tasks, and the mapping itself is low‐dimensional (one spectrum to one depth profile), so the learning problem is comparatively data‐efficient [44]. For these three reasons, AI should be regarded not as a unique replacement for classical inversion but as one of several complementary routes, particularly well matched to the physics of NDP.

On this physical foundation, a small but growing body of work has demonstrated the feasibility of each stage of the analysis chain. ANN‐based pulse‐shape discrimination has been shown to separate genuine ^6^Li signals from low‐energy background in solid‐electrolyte measurements, improving the ROC(Receiver Operating Characteristic) curve area by 77% over the conventional rise‐time method; the same study used interpretable‐AI visualization to expose the learned decision boundary, a first step from black‐box models toward physical understanding in NDP spectroscopy. ANN prediction of concentration profiles directly from spectra has been reported on simulated NDP data with R^2^ scores (coefficients of determination) of 0.94–0.99 and has been shown to outperform conventional automated processing. On the inversion side, the PSO‐MLEM hybrid algorithm, validated on NDP measurements, delivers accuracy better than or comparable to MLEM, basic PSO, and singular‐value‐decomposition least squares, and ANN‐assisted numerical modelling of an NDP system has reported simulated quantification errors within 1% for lithium‐deposition detection and coverage [91]. Beyond NDP itself, closely related machine‐learning work on neutron and x‐ray reflectivity points in the same direction: transformer‐based models now extract thin‐film structures of energy materials directly from reflectivity data [92], reinforcement learning has been used to follow transient phenomena in thin films [93], and neural‐posterior estimation provides fast reflectometry inversion with calibrated uncertainties [94], alongside physics‐informed reflectivity networks [95], gradient‐descent inversion [96], and deep‐unfolding or generative approaches to neutron spectrum unfolding [97]. Because these methods address inverse problems of the same form as NDP (and, for thin‐film energy materials, the very same sample class), they are directly transferable to NDP with modest adaptation. None of these demonstrations, however, has yet been deployed on large‐scale operando battery studies, and most rely on simulated data; their collective significance is to establish feasibility rather than maturity.

The above studies demonstrate the efficient inversion capability of artificial neural networks from spectra to depth distributions within a single NDP technique. However, as described in Section 4.1, complete resolution of the electrode process requires cross‐technology data collaboration. A concrete illustration of what such collaboration means in practice is the combined use of NDP and Rutherford backscattering spectroscopy (RBS) on LiCoO_2_+C_60_ hybrid cathodes (Figure 9) [98]. When performing depth profiling at the same region and scale, NDP can accurately quantify the near‑surface enrichment behavior of lithium, while RBS simultaneously resolves the depth distribution of cobalt, carbon, oxygen, and even current‑collector metals. This work demonstrates that the lithium distribution provided by NDP must be placed in the context of the distribution of the other elements to be fully interpreted: the C_60_‑phase‑induced lithium surface enrichment is essentially a self‑organizing phenomenon driven by the difference in electrochemical potential of the different components. This NDP+RBS combination is, to date, the most complete implementation of the data layer in multimodal characterization with NDP, and it is the natural starting point for the design loop that we now make explicit. The concept is a closed loop of four steps, with NDP at its measurement core. In the first step, operando NDP supplies quantitative, isotope‑specific ^6^Li depth profiles, information that no other probe provides in the same form. In the second step, physics‑informed machine learning inverts these profiles and connects them to degradation mechanisms such as dead‑lithium accumulation, dendrite onset, and interface evolution, with quantified uncertainty. In the third step, the interpreted lithium signatures serve as features and targets for surrogate models that predict the outcome of candidate designs (e.g., electrolyte composition, interface engineering, or cycling protocols) before they are built; fast physics‑informed battery surrogates of this kind already exist, such as parameter‑embedded neural operators and physics‑informed composite networks for cell‑level simulation [99]. In the fourth step, the predicted design is fabricated, cycled, and measured again by NDP, closing the loop and progressively refining the models. The closest existing realizations of the remaining parts of this loop are the lithium‑battery digital twin that couples four‑dimensional modelling with electrochemistry, neutron, and ion‑beam techniques; the latent‑space alignment of heterogeneous material descriptors in multimodal networks [100]; and closed‑loop fast‑charging optimization, which realizes the third step without NDP. No study has yet closed the entire loop with NDP. That is precisely the roadmap proposed here. It should also be stressed that NDP alone cannot describe the complete battery state: it provides the quantitative lithium information, while thermal, mechanical, structural, and electrochemical states are supplied by complementary measurements, a division of labor that the loop makes explicit.

**FIGURE 9:**
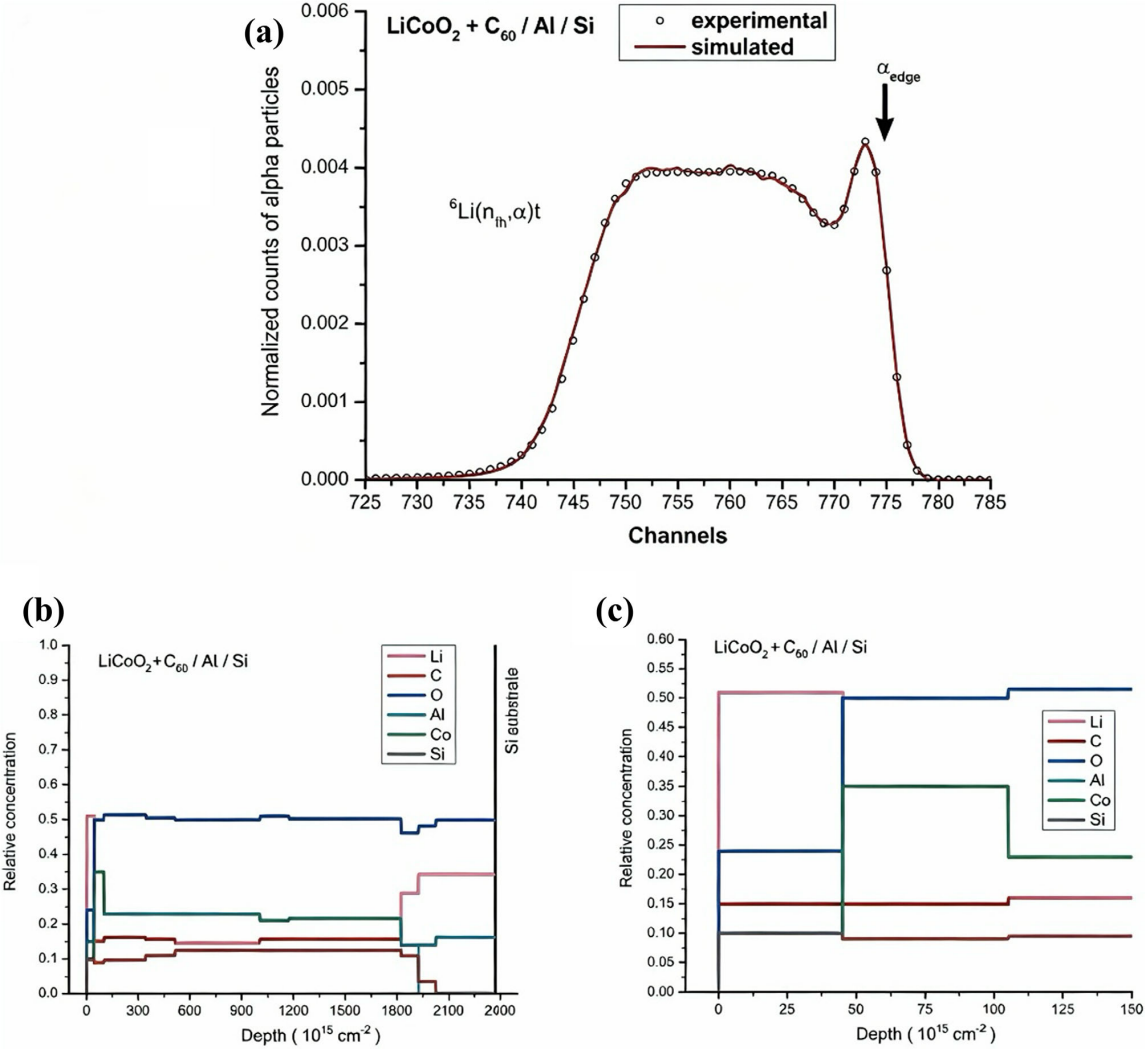
Neutron Depth Profiling Quantification of C_60—_Induced Li Near‐Surface Enrichment and Correlated Multi‐Element Depth Distributions inLiCoO_2_+C_60_ Hybrid Cathodes. (a): NDP spectrum with an optimized simulation (by SIMNRA) of the {LiCoO_2_+C_60_/Al/Si substrate} thin hybrid film with a single bottom current collector (only alpha part of the spectrum was analyzed; energy of **α**_edge_ →2055 keV, energy per channel→2.72 keV/Ch), (b): evaluated depth profiles of structural elements (their relative concentration VERS depth in at. 1015 per cm^2^), (c): details of the profiles in the subsurface layer (0—150 × 1015 at. cm^−2^). Reprinted from Applied Radiation and Isotopes 225, 111980 (2025), with permission from Elsevier [98].

Closing the loop requires progress on four fronts, each a concrete research problem rather than a solved issue. The first is data. There are no public, standardized NDP benchmark datasets, and establishing them simulated and experimental is the enabling step for reproducible machine learning. The second is interpretability and uncertainty. The interpretable‐AI demonstration is only a first step, and uncertainty quantification must be propagated through the inversion and across the loop if design decisions are to rest on NDP‐derived quantities. The third is generalization. Models trained on one chemistry or cell format rarely transfer to another, and physics‐embedded architectures or transfer learning from the reflectivity and spectrum‐unfolding literature will be needed; here, too, cell designs purpose‐built for operando NDP measurement of liquid‐electrolyte batteries [101] will expand the training‐data frontier. The fourth is integration. The physical constraints identified in Section 4.2 (temporal alignment, spatial registration, calibration propagation, and mutual interference) apply directly to any NDP‐in‐the‐loop platform. A realistic sequence of milestones therefore begins with standardized data formats and benchmark datasets, proceeds through physics‐informed inversion with the response matrix embedded and transfer learning from related neutron techniques, continues with a proof‐of‐concept NDP‐in‐the‐loop optimization of a single design parameter, and culminates in an NDP‐enabled digital twin for lifetime prediction. In this way, the promise of NDP as the link between the microscopic mechanisms of lithium‐ion batteries and intelligent design will be realized not as a slogan but as a defined sequence of experiments and models.

## 5 Challenges and Prospects: Advancing NDP Technology to Maturity

NDP is a key tool for revealing internal processes in batteries and has become a powerful technique for understanding critical processes such as lithium transport and interface evolution within batteries. However, it still faces multiple technical bottlenecks in meeting the research and development needs of next‐generation high‐performance batteries. In the future, a collaborative breakthrough is needed through technological innovation, AI‐driven integration, and engineering implementation to ultimately build an intelligent integrated research and development platform (as shown in Figure 10).

**FIGURE 10:**
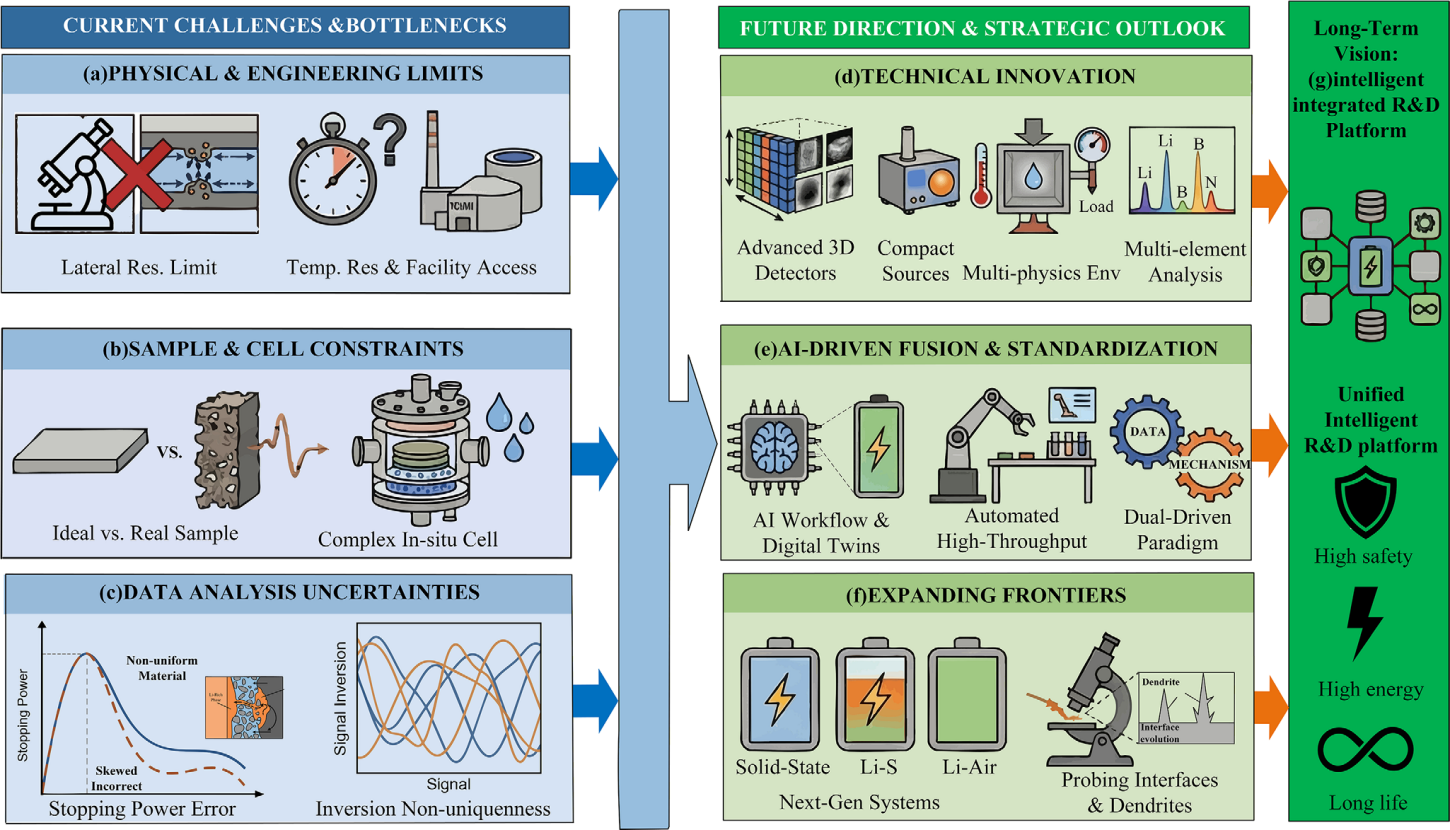
Strategic road map for the advancement of NDP technology in lithium battery research, from current challenges to future prospects. (a): Physical & engineering limits, (b): Sample & cell constraints, (c): Data analysis uncertainties, (d): Technical innovation, (e): AI‐Driven fusion & standardization, (f): Expanding frontiers, (g): Long‐term vision: Intelligent integrated R&D platform.

### 5.1 Current Major Challenges

#### 5.1.1 Physical and Engineering Limitations: Dual Constraints of Spatiotemporal Resolution and Facility Accessibility

From a technical perspective, the lateral resolution of NDP is limited by the neutron beam spot size and detector pixel size. Current conventional systems have a lateral resolution of only hundreds of micrometers, while the scale of critical structures in batteries, such as the SEI film (typically 10–100 nm thick) and dendrite nucleation points (nanometer scale), is orders of magnitude smaller [102]. This disparity prevents direct observation of these core interfacial processes that determine battery safety and lifespan, necessitating a precarious reliance on speculative indirect inferences.

In terms of temporal resolution, the dynamic processes of battery charging and discharging (such as millisecond‐level responses of lithium intercalation/deintercalation and transient evolution of the SEI film) require high‐temporal‐resolution measurements. However, such measurements depend on globally scarce high‐flux pulsed neutron sources. These facilities are extremely limited in number (only about a dozen worldwide), and competition for beam time is fierce, leaving most researchers facing long application cycles and low approval rates. Simultaneously, steady‐state neutron sources only offer second‐level temporal resolution, completely inadequate for capturing the rapid dynamic behavior of batteries, further hindering in‐depth research into dynamic processes [25]. Furthermore, the geographical distribution of large neutron source facilities is extremely uneven, with most developing countries and small and medium‐sized research institutions lacking access, greatly limiting the global dissemination of NDP technology.

#### 5.1.2 Sample and Battery Constraints: The Contradiction Between Ideal Assumptions and Real Operating Conditions

The quantitative analysis model of NDP is based on the assumption of an “ideal uniform thin‐layer sample”, but actual battery electrodes exhibit porous, multiphase, and non‐uniform microstructures: the spatial distribution of active particles, binders, and conductive agents shows significant heterogeneity, and the diffusion path of lithium within the electrode is complex and non‐uniform [103]. This non‐uniformity induces a signal smearing effect that over smooths the detected lithium concentration profile. This results in quantitative errors on the order of tens of percent, making it impossible to accurately reflect the true state of local lithium enrichment or depletion.

The design of in situ batteries present a fundamental engineering paradox: to ensure neutron penetration, the battery casing must use low‐atomic‐number materials such as Kapton and aluminum foil, but at the same time, it must meet multiple operating requirements such as electrochemical stability, airtightness, and temperature control. If a simplified design is adopted, it is impossible to simulate the real current density and thermal management conditions of commercial batteries; pursuing a complex design increases the neutron scattering background, reduces the detection sensitivity of the lithium signal, and introduces additional interface interference, leading to test results that deviate from the actual battery behavior.

#### 5.1.3 Uncertainty in Data Analysis: Inherent Limitations of Dynamic Systems and Inverse Problems

Batteries are typical multi‐layered dynamic complex systems, where the chemical composition and density of each layer, such as the anode, SEI film, electrolyte, and cathode, change dynamically during cycling. The depth calibration and concentration quantification of NDP depend on the “stopping power” of the material, i.e., the rate of energy loss of the emitted charged particles (^3^H and ^4^He) in the material—not of the neutrons, which deposit negligible energy through electronic interactions. Existing databases can only provide static, ideal stopping power values for materials. However, the error in estimated values during dynamic processes can reach 15%–30% [24], directly leading to systematic deviations in depth and concentration. For example, the depth position of lithium may be misjudged by tens of nanometers, and the deviation in concentration quantification may mask critical interface lithium enrichment phenomena.

Furthermore, the energy spectrum inversion of the lithium depth distribution is a mathematically ill‐posed inverse problem: when the signal‐to‐noise ratio is low (such as weak signals from thin interface layers), or the lithium distribution is complex (such as multiple interfaces, local lithium enrichment), different depth distributions may fit similar energy spectra, precipitating the non‐uniqueness of solutions in the mathematical inversion. For example, the lithium concentration distribution of a thin SEI film may be misjudged as fluctuations in bulk lithium. It is necessary to combine other techniques such as transmission electron microscopy (TEM) and x‐ray photoelectron spectroscopy (XPS) for cross‐validation, which not only increases the complexity and cost of the analysis but also reduces the credibility of the results.

Collectively, the above mentioned physical hardware constraints, practical cell sample barriers, and inversion related uncertainties do not merely represent isolated technical defects. They constitute major bottlenecks limiting real world deployment of the NDP centered measurement analysis design closed loop outlined in Section 4. Even promising multimodal fusion and physics informed machine learning workflows cannot fully compensate for fundamental experimental limitations originating from neutron beam facilities, electrode heterogeneity, and ill posed inversion problems.

### 5.2 Future Development Trends and Outlook

#### 5.2.1 Technological Innovation: Toward Higher Spatial and Temporal Resolution

NDP innovation will first surmount physical resolution limits. Advanced three‐dimensional position‐sensitive detectors and compact pulsed neutron sources, integrated with neutron‐focusing optics, will refine lateral resolution to the sub‐micrometer scale and improve temporal resolution toward the millisecond regime, thereby permitting direct visualization of SEI nanostructures and dendrite nucleation sites while capturing transient dynamics [104]. However, the achievable temporal resolution depends on the neutron flux, irradiated area, detector efficiency, lithium concentration, and the counting statistics required for a given uncertainty, and millisecond time resolution is not yet demonstrated with current steady‐state facilities which typically operate at second‐level resolution. In parallel, a multi‐physics in situ platform that couples temperature, pressure, and electrochemical control with multimodal probes, such as x‐ray computed tomography and Raman spectroscopy—will deliver correlated multidimensional information. This integration will overcome the current restriction to single‐element quantification and supply direct experimental guidance for next‐generation battery materials [27].

#### 5.2.2 AI‐Driven Integration and Standardization: From “Trial and Error Iteration” to “Rational Design”

AI will profoundly reshape the NDP research paradigm. Deep learning and Bayesian inference will resolve spectral inversion ambiguities, while digital twins grounded in computational materials science will enable multi‐scale performance predictions, driving the transition from trial and error iteration to rational design. Standardization and automation will proceed in tandem: unified measurement protocols and automated integrated systems will forge a dual‑driven closed loop of experimental observation, data mining, mechanistic analysis, and model prediction, sharply shortening R&D cycles and accelerating the translation of fundamental insights into practice.

Standardization efforts should not be restricted to well‐defined model thin film cells; benchmark datasets covering heterogeneous porous practical electrodes are also urgently required to narrow the gap between proof of concept demonstrations and real world battery research, as emphasized in Section 3.5.

#### 5.2.3 Application Expansion and Engineering Implementation: From the Laboratory to Industry

NDP technology will continue to expand to new‐generation battery systems such as solid‐state, lithium‐sulfur, and lithium‐air batteries, focusing on key scientific problems such as “lithium distribution evolution at the solid electrolyte/electrode interface” and “dendrite nucleation and growth,” providing direct experimental evidence for the design of high‐safety, high‐energy‐density batteries [105, 106]. On the engineering side, the most promising route is to couple NDP with the intelligent‐design loop outlined in Section 4.3, improving the consistency and reliability of battery production. The long‐term vision is an intelligent integrated R&D platform in which NDP serves as the quantitative lithium‐measurement core [107]. It requires step‐by‐step breakthroughs across instrumentation, sample‐cell engineering, data‐analysis methodology, and multi‐source data fusion.

By integrating in situ testing, multimodal characterization, AI analysis, and digital twin technology, we aim to achieve an end‐to‐end closed loop from material design to battery performance optimization. This will fundamentally address battery safety hazards, energy density bottlenecks, and lifespan degradation issues, supporting the large‐scale application of high‐safety, high‐energy, and long‐life batteries.

## 6 Conclusion

Neutron Depth Profiling (NDP) leverages the ultra‐high neutron capture cross section of ^6^Li to deliver non‐destructive, operando analysis with two reasonably distinctive characteristics: isotope specific recognition of ^6^Li and quantitative depth‐dependent lithium concentration profiling. It enables real‐time tracking of lithium redistribution inside working batteries. This capability bridges macroscopic electrochemical performance and microscopic ion behavior, circumventing the limitations of cyclic voltammetry and impedance spectroscopy while eliminating the destructive artifacts of electron microscopy. NDP has thus become a valuable tool for deciphering lithium transport, interfacial evolution, and degradation mechanisms. Its seminal achievements include revealing non‐uniform lithium nucleation in solid electrolytes and precisely quantifying the spatial distribution of inactive (“dead”) lithium. Combined with complementary characterization techniques that confirm the electrochemical inactivity of such lithium, this quantification further enables evaluation of its contribution to capacity fade. This work also uncovers reversible short‐circuit phenomena that challenge conventional diffusion‐limited paradigms. These insights have transformed interface engineering from passive mitigation of deficiencies into the active design of controllable lithium dynamics.

Instrument advances ranging from global steady‐state neutron facilities to position sensitive N4DP detectors have extended NDP from one dimensional depth profiling toward two and three dimensional lithium mapping. Meanwhile, inversion workflows have evolved from classical linear solvers toward hybrid intelligent algorithms including PSO MLEM and artificial neural networks. This review further highlights an integrated multimodal monitoring framework for intelligent battery design: NDP supplies the isotope resolved quantitative lithium depth information as the core measurement input; multimodal complementary measurements provide thermal, structural, and electrolyte state observables; and physics‐informed machine learning interprets multi‐source datasets to infer degradation mechanisms and guide candidate material cell design. It should be emphasized that most integrated closed loop workflows remain at the proof of concept stage rather than mature experimental reality.

Importantly, NDP cannot provide complete elemental distributions across battery samples, nor can it resolve nanoscale interface structure or morphology. As a relatively little known characterization technique, NDP possesses specific core strengths alongside substantial practical limitations. Operando NDP battery measurements are technically imperfect and not yet fully mature, and access to high‐flux neutron beam facilities remains highly limited. Furthermore, spectral reconstruction models for deriving lithium depth profiles are subject to multiple sources of uncertainty.

Moving forward, standardized benchmark datasets, physics aware machine learning pipelines, and improved operando cell architectures will accelerate translation from laboratory model cells toward practical high energy density battery systems including solid state, lithium sulfur and lithium air chemistries. Realizing the full potential of NDP as the core link between microscopic mechanisms and intelligent battery design requires continuous technical improvement and tight synergy with complementary characterization tools and physically grounded modelling. These advance will facilitate the development of next‐generation high safety and high energy density batteries for electric vehicle and grid scale energy storage scenarios.

## Author Contributions

**Yulong Zhang**: writing – original draft, project administration. **Chunjing Yue**: writing – review and editing, supervision. **Shijiao Liu**: writing – review and editing, conceptualization. **Muyang Chen**: writing – review and editing, conceptualization. **Xiaokang Shi**: formal analysis, visualization. **Rui Tan**: conceptualization, visualization. **Hongqing Hao**: conceptualization, project administration. **Jie Yang**: conceptualization, visualization. **Xinhua Liu**: writing – review and editing, resources.

## Conflicts of Interest

The authors declare no conflicts of interest.

## Data Availability

The data that support the findings of this study are available from the corresponding author upon reasonable request.
